# Effects of projected increases in heat exposure on linguistic development in two-year-old children: A longitudinal modified treatment policy analysis

**DOI:** 10.1097/EE9.0000000000000423

**Published:** 2025-10-13

**Authors:** Guillaume Barbalat, Ariane Guilbert, Marie-Aline Charles, Ian Hough, Ludivine Launay, Itai Kloog, Johanna Lepeule

**Affiliations:** a Université Grenoble Alpes, INSERM U1209, CNRS UMR 5309, Institut pour l’Avancée des Biosciences (IAB), Team of Environmental Epidemiology Applied to Development and Respiratory Health, 38000 Grenoble, France; b Centre ressource de réhabilitation psychosociale et de remédiation cognitive, Hôpital Le Vinatier, Pôle Centre rive gauche, UMR 5229, CNRS & Université Claude Bernard Lyon 1, Lyon, France; c Institut National d’Études Démographiques (Ined), Institut National de la Santé et de la Recherche Médicale (Inserm), Établissement Français du Sang (EFS), Étude Longitudinale Française depuis l’Enfance (ELFE) Joint Unit, Paris, France; d Université Grenoble Alpes, CNRS, INRAE, IRD, INP-G, IGE (UMR 5001), Grenoble, France; e U1086 Institut National de la Santé et de la Recherche Médicale (Inserm) Anticipe, Caen Cedex, France; f University Hospital of Caen, Caen Cedex, France; g Plateforme MapInMed, US PLATON, Caen Cedex, France; h The Department of Environmental, Geoinformatics and Urban Planning Sciences, Ben-Gurion University of the Negev, Beer Sheva, Israel; i Department of Environmental Medicine and Public Health, Icahn School of Medicine at Mount Sinai, New York City, New York

## Abstract

**Background:**

Previous studies have demonstrated that in utero and early life heat exposure can influence neurodevelopment. However, to our knowledge, these investigations have not evaluated realistic counterfactual scenarios; instead, they have primarily relied on static, crude comparisons of extreme temperatures versus a reference temperature over an extended period.

**Methods:**

We employed the framework of longitudinal modified treatment policy to examine the impact of heat exposure during prenatal and postnatal periods on the linguistic development of two-year-old children in the Etude Longitudinale Française depuis l’Enfance birth cohort (N = 12,163). Heat exposure was defined as the number of periods when overall daytime and nighttime daily temperatures surpassed the 90th percentile (20.6, 27.5, and 15.3 °C, respectively) for at least two consecutive days. Context-specific counterfactual scenarios were constructed by increasing daily temperatures by 1, 2, or 3 °C, in line with projections from Intergovernmental Panel on Climate Change scenarios. Causal effects were estimated by comparing the population mean outcomes under hypothetical counterfactual scenarios vs. those actually observed in the data using a doubly robust estimation technique (targeted maximum likelihood estimation). A library of machine learning algorithms was employed to model the intricate relationships between covariates and both the exposure and outcome variables.

**Results:**

In counterfactual scenarios where daily temperature increases by one degree, mean differences in log-transformed population outcome did not reach statistical significance. A two-degree daily increase in nighttime temperature showed a decrease in linguistic development scores of 30% (*P* < 0.001). A three-degree increase in overall, daytime and nighttime daily temperatures showed a decrease in scores of at least 6% (*P* < 0.003).

**Conclusion:**

Our study revealed a negative impact of increased air temperatures on the linguistic development of 2-year-old children in counterfactual scenarios involving two- and three-degree temperature rises. The longitudinal modified treatment policy approach offers valuable new insights for causal inference in environmental epidemiology, particularly through its ability to directly assess the effects of anticipated, policy-relevant temperature changes.

**What this study adds**

We examined the impact of heat exposure during prenatal and postnatal periods on the linguistic development of 2-year-old children recruited in the Etude Longitudinale Française depuis l’Enfance birth cohort (N = 12,163). Previous studies investigating the effect of temperature on neurodevelopment relied on crude static comparisons of extreme temperatures versus a reference temperature over a prolonged period. Instead, our study examined the effect of realistic counterfactual scenarios based on projections from the Intergovernmental Panel on Climate Change. It therefore provides insights that are directly relevant for public health policy and climate adaptation. Here, we showed an adverse impact of two- and three-degree temperature increases on linguistic development scores.

## Introduction

Numerous studies have demonstrated that exposure to extreme temperatures is associated with a wide range of adverse health outcomes, including respiratory and cardiovascular diseases, mortality, and adverse perinatal, childhood, and maternal health outcomes.^1–8^ Neurodevelopmental changes as a result of extreme temperatures have also been recently demonstrated. Acute exposure to high temperatures has been related to cognitive dysfunctions in children and adolescents, such as difficulties with attention-demanding tasks and decreased academic achievements;^9,10^ increased mental-health-related Emergency Department visits;^11^ and crisis help-seeking behaviors.^12^ Longer-term temperature exposures have also been associated with adverse neurodevelopmental outcomes. In utero heat exposure has been associated with psychiatric disorders later in life;^13^ heat exposure in the previous 2 months has been related to increased attention problems in adolescents;^14^ and rising average temperatures over the preceding 1–3 years have been related to increased aggressive behaviors in children aged 9–18.^15^ Finally, we recently showed that heat exposure in both the pre- and postnatal periods were responsible for impairments in linguistic development.^16^

To provide reliable effect estimates and in turn inform effective policy and public health interventions, these studies have primarily focused on collecting comprehensive, population-specific datasets with a large number of observations, utilizing health records or large cohorts, and developing highly resolved exposure maps to minimize measurement error. But while they have provided valuable insights into the effects of temperature on neurodevelopment, their exposure scenarios often lacked real-world relevance. Typically, these studies compare the impact of extreme temperatures (such as the 95th percentile of a given temperature distribution) to a reference value (like the median), effectively defining two static counterfactual scenarios in which individuals are exposed to a fixed temperature level throughout the study period. However, it is unrealistic to assume that an individual would be exposed to constant extreme or average temperatures throughout pregnancy and early childhood, even when accounting for possible acclimatization within specific regions. Some studies report lag-specific associations (i.e., effects observed in specific time windows), but these analyses assume that exposures in all other periods remain constant, an assumption that is unlikely to hold.

Other studies have investigated risk increases associated with incremental rises in temperature, although, to our knowledge, not within a neurodevelopmental context (e.g., investigating low birth weight).^17^ Typically, these studies have applied standard linear modeling, which assumes a constant, linear effect across the entire exposure range. In other words, they posit that a one-unit increase in temperature produces the same average effect regardless of where it occurs on the temperature scale. Such a predefined linear assumption can be considered overly simplistic, while nonlinear approaches may provide a more accurate representation of the exposure-response relationship. However, investigating causality through modeling the exposure-response relationship (linearly or not) encounters two main challenges. First, it frequently requires extrapolation beyond the observed data range, which may result in modeling relationships that lack strong empirical support. Second, counterfactual scenarios derived from such models inherently rely on static comparisons, which, as noted earlier, are often unrealistic.

A more relevant research question would investigate the effects of temperature increases, such as those projected by the Intergovernmental Panel on Climate Change (IPCC),^18^ rather than relying on static hypothetical exposures. Such an analysis could yield actionable evidence directly tied to scientific climate projections, offering policymakers and communities reliable guidance for mitigating developmental risks in vulnerable populations. Standard analytical methods, which contrast fixed exposure scenarios, are ill-suited for this purpose. In this study, we applied a recent methodological approach that overcomes the limitations of static exposure models by estimating the effect of heat exposure from conception to age two on linguistic development, using realistic scenarios based on IPCC projections. This method employs the “longitudinal modified treatment policy” (LMTP) framework, defining the causal parameter as the contrast between counterfactual outcomes under a modified exposure (e.g., increased temperatures) and the observed factual outcomes. Heat exposure was defined as the number of periods during which daily temperatures exceeded the 90th percentile for at least two consecutive days. Context-specific counterfactual scenarios were generated by increasing daily temperatures by 1, 2, or 3 °C, consistent with IPCC scenario projections. We used the same dataset as in our previous publication,^16^ allowing for a direct assessment of the added value these innovations provide.

## Methods

### Data

#### Database

We relied on the large French prospective birth cohort named Etude Longitudinale Française depuis l’Enfance (ELFE), launched in 2011 in 344 maternity units.^19^ Recruitment took place during 25 selected days in 2011, spread over four periods throughout the year. Each of the four waves included between 1,819 and 3,722 participants.

Exclusion criteria were: multiple births of more than two children; underaged parents or parents who were not capable of giving informed consent; planning to leave metropolitan France within the next 3 years; inability to read/understand French, Arabic, Turkish, or English; and children born before 32 weeks of amenorrhea. Indeed, the ELFE cohort was designed to study child development and health in the general population. Very preterm infants (<32 weeks) and multiple births of two (or more) children have distinct medical trajectories and risks (e.g., neonatal complications and prolonged Neonatal Intensive Care Unit stays) that could distort the general patterns of interest and require specialized follow-up. Including them would have introduced substantial heterogeneity that was not consistent with the objectives of ELFE.

Around 51% of the mothers who met the inclusion criteria agreed to participate, and 18,329 children were enrolled. For the purpose of this study, we also excluded mothers with multiple gestations (N = 576). We intentionally chose not to exclude preterm infants for two reasons: (1) prematurity may lie on the causal pathway between prenatal heat exposure and neurodevelopmental outcomes;^20^ and (2) excluding preterm births could introduce collider-stratification bias, as prematurity may act as a collider between temperature and other environmental exposures that influence neurodevelopment. Consequently, preterm infants were retained in our analyses.

Demographic, socioeconomic, and lifestyle information, as well as information on the child’s development were collected by questionnaire at each survey wave at the maternity unit (face to face interviews conducted during four recruitment periods: 1–4 April, 27 June–4 July, 27 September–4 October, and 28 November–5 December) and during regular follow-ups of the children (telephone interviews). For the purpose of this study, we used follow-up information collected at 2 months, 1 year, and 2 years.

#### Outcome variable: linguistic development

Our outcome variable was the total score from the vocabulary production checklist of the MacArthur-Bates Communicative Development Inventories (MB-CDI), an index of linguistic development.^21^ Vocabulary production was assessed by asking parents to state whether their 2-year-old child is able to pronounce spontaneously each word out of a list of 100 words. We then calculated the total score as the number of words correctly pronounced by the child (higher scores indicate better linguistic development). MB-CDI scores were log-transformed using log(x+1), facilitating interpretation in terms of percentages. Scores were obtained from the mother’s interview. If absent, we used scores reported by the father (N = 104).

#### Exposure to ambient heat

Exposures were based on air temperatures from conception to 2 years of age. ELFE excluded children born before 32 weeks of amenorrhea. To ensure consistent exposure assessment across children with varying gestational ages, and considering that preterm children born as early as 30 weeks of gestation were included, we excluded prenatal exposure data occurring after the 30th week of gestation for all participants. Since linguistic development testing began at 23 months postnatally, we excluded exposure observations beyond 23 months of age. Moreover, we excluded exposure data past the 21st month postpartum, reasoning that the final weeks of exposure would minimally impact linguistic development processes. We chose to restrict the postnatal exposure window to the first 90 weeks of life, divided into nine 10-week periods, to avoid overlap with the timing of outcome assessment, which began as early as 23 months (approximately 100 weeks). Including a tenth window (weeks 91–100) would risk capturing exposures whose potential neurodevelopmental effects would not yet be observable at the time of language assessment, due to potential delays between environmental insults and their effects on cognitive outcomes in early life.^22^ This approach ensures temporal precedence and reduces the likelihood of exposure misclassification relative to outcome onset. Pre- and postnatal exposures were divided into 12 periods of 10 weeks each, covering a total of 120 weeks of exposure data (as a compromise between temporal precision and model estimability).

Although data from monitoring stations were available during the study period, these stations were not necessarily located near the participants’ actual residences. Therefore, we used estimated daily ambient temperatures at the mother’s and child’s home addresses obtained from highly-resolved spatiotemporal modeling. Exact home addresses (including changes of residence during pregnancy and childhood) were geocoded using a parcel-level database. The geospatial model used a multi-stage ensemble approach combining three basis learners (among which linear mixed models, random forests, and gradient boosting) to calibrate temperature measured at monitoring stations with spatiotemporal predictors.^23^ Predictions of ambient temperature were estimated from 2000 to 2018 at a 1 km spatial resolution across France and at a 200 m spatial resolution over urban areas with >50,000 inhabitants. Three indicators of temperature were calculated: minimum (Tmin), maximum (Tmax), and mean (Tmean) air temperature, taken as proxies for nighttime, daytime, and overall temperatures, respectively, since the minimum temperature typically occurs during the night and the maximum typically occurs during daylight hours. Daily Tmin was predicted using the lowest ambient temperature recorded hourly at the nearest temperature station from 18:00 UTC the previous day until 18:00 UTC on the day of interest; daily Tmax was predicted using the highest ambient temperature recorded at temperature station from 6:00 UTC on the day of interest until 6:00 UTC the following day; and Tmean was predicted using the mean of all (at least 24) ambient temperatures from 0:00 UTC on the day of interest until 0:00 UTC the following day. In terms of model performance, the mean cross-validated R2 were higher than 0.9. For this study, we used predicted temperature data only for the period relevant to the portion of the ELFE study and focused on, namely, 2011–2013.

We defined hot days within each 10-week period as those exceeding predefined high temperature percentiles. Given the relatively mild temperatures in 2010 and 2011, we focused on heat spikes rather than heat waves. Météo-France defines a heat spike as a brief (24–48 hours) period of temperatures significantly above seasonal averages, distinct from longer-lasting heat waves.^24^ Our heat exposure measure counted the number of periods in which daily temperatures exceeded the 90th percentile threshold (Tmean = 20.6 °C, Tmax = 27.5 °C, and Tmin = 15.3 °C) for at least two consecutive days. As sensitivity analyses, we examined scenarios in which heat exposure was defined by the number of instances where daily temperatures surpassed the 80th (Tmean = 18.2 °C, Tmax = 24.6 °C, and Tmin = 13.2 °C) and 85th (Tmean = 19.3 °C, Tmax = 25.9 °C, and Tmin = 14.1 °C) percentiles. Note that there were too few observations with temperatures above the 95th percentile; therefore, we decided not to use this cutoff as the threshold for heat exposure.

#### Covariates

Covariates are described in detail in the Supplementary Method; https://links.lww.com/EE/A374. Briefly, main covariates included variables linked to the socioeconomic context and urbanization of the neighborhood; vegetation; socioeconomic status and demographic indicators directly related to the parents; the number of languages spoken at home; variables linked to prepregnancy medical history: parity, neurodevelopmental disorders, maternal obesity; food and drug exposure during pregnancy and after birth; age and sex of the child. These variables were all assessed at a single time point. In addition, cold was used as a time-dependent confounder.^25^

#### Missing data

From the original 17,753 single pregnancy observations, 4,697 were lost due to withdrawal or lost to follow-up. Among the remaining participants, we excluded 515 participants with missing values for the MB-CDI and 289 further participants with missing exposure data. We then excluded participants with more than 30% missing values in the covariates (N = 89) to balance the need for sufficient information per individual with the goal of retaining an adequate sample size for analysis. The final dataset comprised N = 12,163 participants. Finally, missing data in the covariates were imputed with the mice R package (using single imputation). Imputation relied on prediction from mean exposures, covariates, and outcome.

### Analysis

Our analysis used the framework of LMTP.^26^ LMTP is a recent advancement in causal inference that enables researchers to define and estimate the effects of exposures that vary over time. Unlike traditional approaches that rely on static or fixed interventions, LMTP allows for more realistic hypothetical scenarios by modifying exposures relative to their naturally observed values, making the results more directly applicable to real-world policy questions. The LMTP approach has been applied in various settings.^27–30^ However, to our knowledge, LMTP has been used only once in environmental research: in a brief demonstrative example examining the impact of a policy aimed at reducing nitrogen dioxide concentrations on asthma symptoms in children aged 6–11 years.^31^

#### Counterfactual outcomes and causal parameter of interest

Counterfactual exposures were based on the “low emissions” and “intermediate” IPCC scenarios, where global mean temperatures are projected to increase by up to 3.5 °C by 2100.^32^ We generated counterfactual outcomes $Y^{d}$ by shifting the observed daily temperature Temp by an additive constant k, where k={1;2;3}, defining a shift function d(Temp,k)=k+Temp. For each child, the counterfactual outcome $Y^{d}$ was defined as his or her MB-CDI score had he or she been exposed to a number of at least two consecutive hot days in each 10-week period where factual daily temperatures Temp were increased by k degrees. Our causal parameter of interest was obtained by comparing mean counterfactual outcome $Y^{d}$ with mean factual, real-world outcome Y: $\theta =E\left[Y^{d}\right]-E\left[Y\right]$

#### Doubly robust estimation

We estimated our causal parameter of interest using a doubly robust nonparametric estimation technique. Double-robustness relies on estimation of (1) the outcome regression (where outcome values are predicted based on our set of covariates) and (2) the exposure mechanism (where probabilities of exposures are predicted based on the covariates). Both estimation steps are integrated by updating (“targeting”) the initial estimation of (1) the predicted outcome, based on (2) probabilities of exposures,^33^ aiming to create a pseudo-random population of observations with respect to the distribution of the covariates. This also optimizes the bias-variance tradeoff for the wished-for causal parameter of interest. Further, it provides a solution to the efficient influence function estimating equation, endowing it with desirable asymptotic properties that enable valid statistical inference under regularity and convergence conditions.^33^

Doubly robust estimators yield unbiased estimates if either the estimated outcome regression or exposure mechanism is consistently estimated.^33^ When both the outcome regression and exposure mechanisms are consistently estimated, doubly-robust estimators are asymptotically efficient.^33^ For the purpose of this study, we used targeted maximum likelihood estimation (TMLE), which guarantees that our bounded parameters remain plausible.^26^

Using TMLE, we first estimated the mean population MB-CDI score E[Y] under a “no intervention” strategy (i.e., under observed exposures), adjusted for the covariates. Second, we estimated mean population MB-CDI scores $E\left[Y^{d}\right]$ under various exposure scenarios (i.e., increasing temperature by one, two, or three degrees), again adjusted for the covariates. Third, we contrasted E[Y] with $E\left[Y^{d}\right]$ to evaluate the potential impact of the hypothetical interventions relative to the observed exposures on the mean population MB-CDI scores.

#### Using machine learning algorithms to handle complex data structures and support double robustness

TMLE leverages machine learning (ML) algorithms to estimate both the outcome regression and the exposure mechanism, accounting for the complex interplay between time-varying exposures, confounders, and outcome processes, and thereby enhancing the likelihood of correct specification in at least one of these models. This approach offers a significant advantage over prespecified parametric models, such as general linear models, which often impose strong assumptions on data distributions.

Of note, the exposure mechanism (probability of experiencing counterfactual exposures) is estimated via so-called “density ratios,” which contrast the probability density functions of the counterfactual and factual exposures, conditional on covariate history.^34^ These density ratios are calculated at each time step. They are akin to stabilized weights in inverse probability of treatment weighting approaches and are applied to the initial outcome prediction during the targeting step.

We used an ensemble ML algorithm called SuperLearner, defined as a weighted linear combination of basic learners obtained through cross-validation and minimization of a loss function.^35^ The following basis learners were used: Linear model with main effects only, Bayesian general linear models, Linear regression with L1-regularization, Multivariate adaptive regression splines, Random Forest, extreme gradient boosting, and a benchmark algorithm that calculates the average (mean) of all outcome values in the training data. Parameters were those applied routinely from the SuperLearner R package. The SuperLearner is implemented at each time step, and the estimation of the outcome regression and the density ratios is conditioned on the complete history of all variables (no Markov assumptions).

#### Identifiability of the causal parameter of interest

As with any causal framework, identifiability depends on two key assumptions: the absence of unmeasured confounders and the positivity assumption. To minimize unmeasured confounding, we included a comprehensive set of covariates, carefully selected to avoid mediators and reverse causation bias, and incorporated cold peaks as time-dependent confounders (Supplementary Method; https://links.lww.com/EE/A374).

The positivity assumption requires that, for any given time slice, every individual in the population has a nonzero probability of receiving each level of the exposure, given their covariate values.^36^ We avoided structural violations of the positivity assumption by considering shifts that were plausible considering the data at hand (i.e., not considering extreme temperature increases projected by high emissions scenarios). To safeguard against practical positivity violations, we verified that each individual had a positive probability of experiencing counterfactual exposure scenarios, given their characteristics. As mentioned above, these probabilities are estimated by comparing the probability density functions of the counterfactual vs. factual exposures, conditional on covariate history, at each time step (density ratios). Extreme ratios indicate practical positivity violations and can inflate the variance of our causal estimate. To mitigate this, we performed our analysis after truncating density ratios exceeding the 99.9th percentile, effectively capping excessively high values at these thresholds.^37^

All statistical analyses were performed in R version 4.0.3. and package *lmtp*.

## Results

### Characteristics of the data

Characteristics of the participants are reported in the Supplementary Table; https://links.lww.com/EE/A374. Adjusting for the covariates, the log-transformed population mean of the MB-CDI under no counterfactual scenario was of 4.196 (95% confidence interval [CI] = 4.186, 4.206). Back-transformed values were of mean=65.4 and 95% CI=[64.8, 66.1].

**Figure 1.**
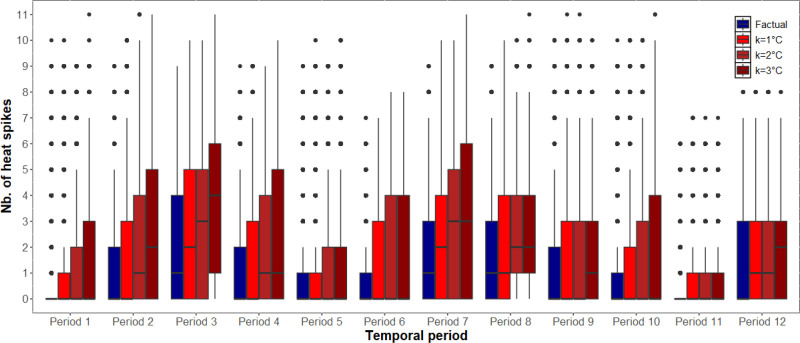
Counterfactual heat exposures based on overall temperature (Tmean) with heat thresholds set at the 90^th^ percentile (Tmean = 20.6 °C). X-axis: Temporal period; Y-axis: Number of heat spikes. Boxplots comparing factual (blue) and counterfactual (red) temperature exposures for each 10-week period. Counterfactual scenarios represent increases of 1, 2, or 3 °C added to observed daily temperatures. Heat exposure was quantified as heat spikes, defined as the number of occurrences in each 10-week period where the daily temperature exceeded the 90th percentile (Tmean = 20.6 °C) for at least two consecutive days. The box represents the interquartile range (IQR), spanning from the first quartile (Q1) to the third quartile (Q3), with the horizontal line inside the box indicating the median. The whiskers extend to the most extreme data points within 1.5 times the IQR from the quartiles, and points beyond the whiskers are plotted individually as outliers.

**Figure 2.**
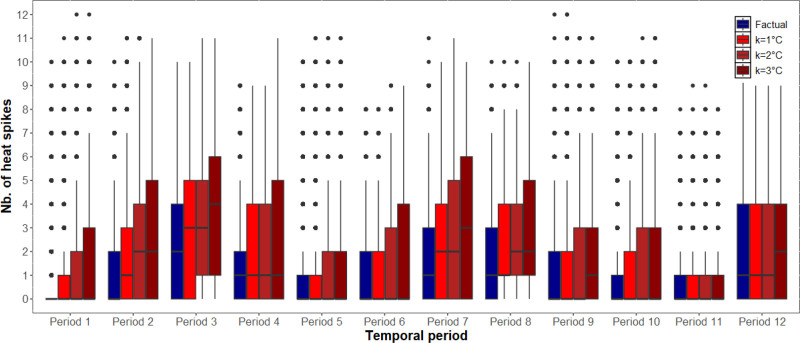
Counterfactual heat exposures based on daytime temperature (Tmax) with heat thresholds set at the 90th percentile (Tmax = 27.5 °C). X-axis: Temporal period; Y-axis: Number of heat spikes. Boxplots comparing factual (blue) and counterfactual (red) temperature exposures for each 10-week period. Counterfactual scenarios represent increases of 1, 2, or 3 °C added to observed daily temperatures. Heat exposure was quantified as heat spikes, defined as the number of occurrences in each 10-week period where the daily temperature exceeded the 90th percentile (Tmax = 27.5 °C) for at least two consecutive days. The box represents the interquartile range (IQR), spanning from the first quartile (Q1) to the third quartile (Q3), with the horizontal line inside the box indicating the median. The whiskers extend to the most extreme data points within 1.5 times the IQR from the quartiles, and points beyond the whiskers are plotted individually as outliers.

**Figure 3.**
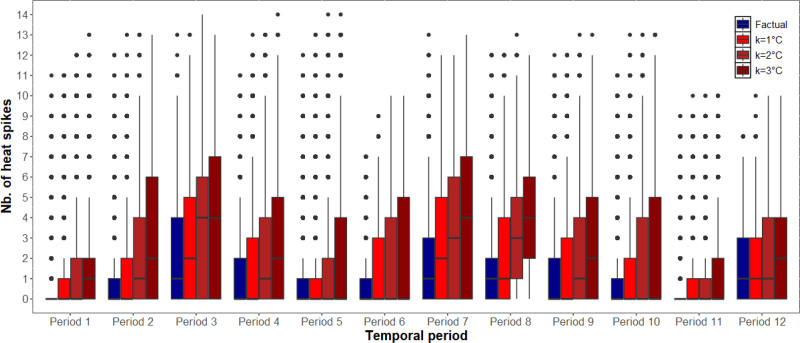
Counterfactual heat exposures based on night-time temperature (Tmin) with heat thresholds set at the 90^th^ percentile (Tmin = 15.3 °C). X-axis: Temporal period; Y-axis: Number of heat spikes. Boxplots comparing factual (blue) and counterfactual (red) temperature exposures for each 10-week period. Counterfactual scenarios represent increases of 1, 2, or 3 °C added to observed daily temperatures. Heat exposure was quantified as heat spikes, defined as the number of occurrences in each 10-week period where the daily temperature exceeded the 90th percentile (Tmin = 15.3 °C) for at least two consecutive days. The box represents the interquartile range (IQR), spanning from the first quartile (Q1) to the third quartile (Q3), with the horizontal line inside the box indicating the median. The whiskers extend to the most extreme data points within 1.5 times the IQR from the quartiles, and points beyond the whiskers are plotted individually as outliers.

Factual and counterfactual daily temperature distributions for Tmean, Tmax, and Tmin are displayed in Supplementary Figure 1; https://links.lww.com/EE/A374. Factual and counterfactual heat exposure distributions aggregated across all 10-week periods are displayed in Supplementary Figures 2–4; https://links.lww.com/EE/A374. Boxplots of factual and counterfactual heat exposures for each of the 10-week periods are shown in Figures 1–3 and Supplementary Figures 5–7; https://links.lww.com/EE/A374. Note the subtle differences between the distributions.

### Density ratio estimation

As a reminder, density ratio estimates are a measure of the exposure mechanism (probability of experiencing counterfactual exposures). Density ratios estimates are displayed for each temporal period in Supplementary Figures 8–10; https://links.lww.com/EE/A374. Truncating density ratios to the 99.9th percentile, we obtained a mean value of 0.90 (median was 0.91), with a maximum value of 24.55.

### Causal estimates across scenarios

**Figure 4.**
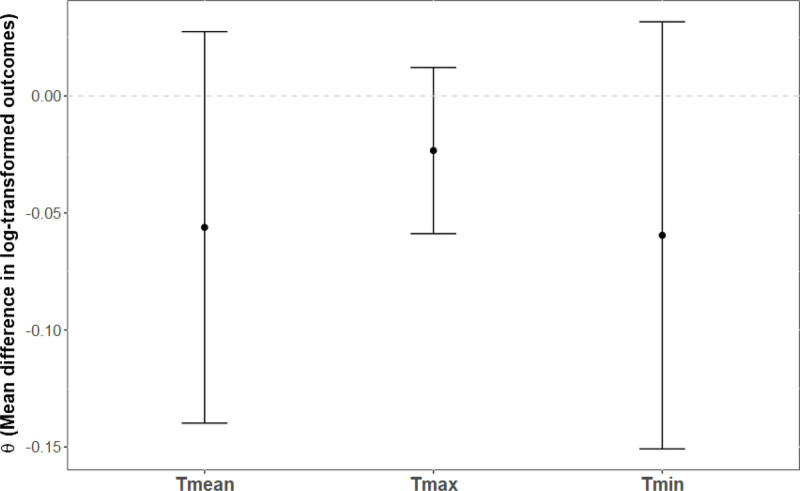
Effects of rising daily air temperatures by one degree Celsius on MB-CDI scores with heat thresholds set at the 90th percentile. X-axis: Temperature indicators; Y-axis: Mean difference in log-transformed outcomes. Mean differences in log-transformed MB-CDI scores are plotted along with 95% confidence intervals for counterfactual scenarios involving increases in daily temperature by 1 °C, with heat thresholds set at the 90th percentile for overall (Tmean = 20.6 °C), daytime (Tmax = 27.5 °C), and nighttime (Tmin = 15.3 °C) temperatures. Estimates shown in red indicate statistical significance. Legend.

**Figure 5.**
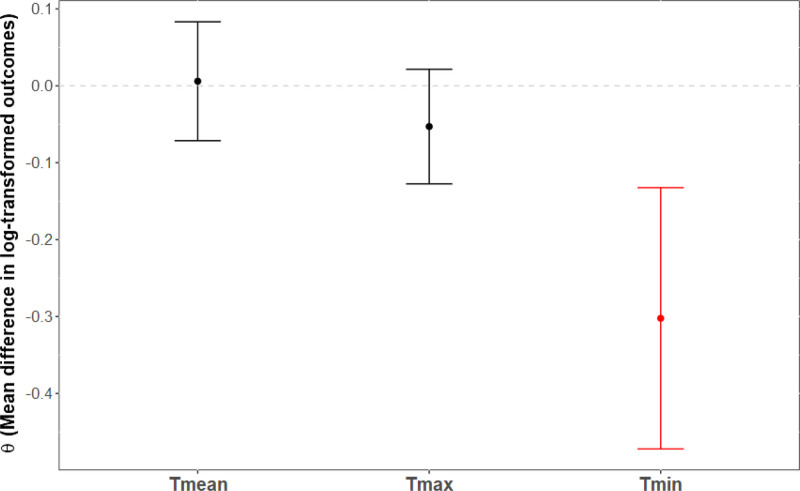
Effects of rising daily air temperatures by two degrees Celsius on MB-CDI scores with heat thresholds set at the 90th percentile. X-axis: Temperature indicators; Y-axis: Mean difference in log-transformed outcomes. Mean differences in log-transformed MB-CDI scores are plotted along with 95% confidence intervals for counterfactual scenarios involving increases in daily temperature by 2 °C, with heat thresholds set at the 90th percentile for overall (Tmean = 20.6 °C), daytime (Tmax = 27.5°C), and nighttime (Tmin = 15.3 °C) temperatures. Estimates shown in red indicate statistical significance. Legend.

**Figure 6.**
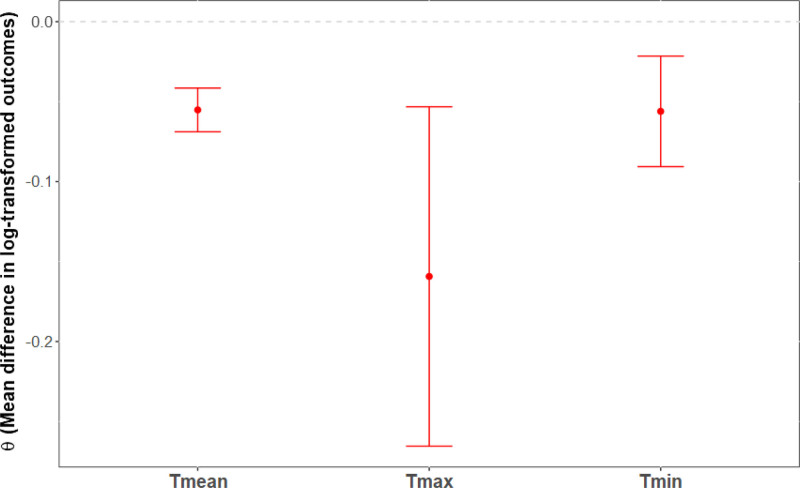
Effects of rising daily air temperatures by three degrees Celsius on MB-CDI scores with heat thresholds set at the 90th percentile. X-axis: Temperature indicators; Y-axis: Mean difference in log-transformed outcomes. Mean differences in log-transformed MB-CDI scores are plotted along with 95% confidence intervals for counterfactual scenarios involving increases in daily temperature by 3 °C, with heat thresholds set at the 90th percentile for overall (Tmean = 20.6 °C), daytime (Tmax = 27.5 °C), and nighttime (Tmin = 15.3 °C) temperatures. Estimates shown in red indicate statistical significance. Legend.

Figures 4–6 display the mean difference in log-transformed MB-CDI scores between counterfactual scenarios and factual observations for Tmean, Tmax, and Tmin with heat thresholds set at the 90th percentiles (Tmean = 20.6 °C, Tmax = 27.5 °C, and Tmin = 15.3 °C), and temperature increases of 1, 2, and 3 °C. In counterfactual scenarios where daily temperature increases by 1 °C (Figure 4), the range of the mean difference in log-transformed population outcome was θ = (−0.06, −0.02]. None of these differences reached statistical significance. In counterfactual scenarios where daily temperature increases by 2 °C (Figure 5), the range of the mean difference in log-transformed population outcome was of θ = (−0.30, 0.006). Nighttime temperature increases resulted in a 30% decrease on the MB-CDI score (θ = −0.30, 95% CI = −0.47, −0.13, *P* < 0.001). Other differences were not significant. Finally, in counterfactual scenarios where daily temperature increases by 3 °C, the range of the mean difference in log-transformed population outcomes was of θ = −0.16, −0.06]. All mean differences were significant, indicating a decrease in the MB-CDI score of at least 6% (θ’s<−0.06, *P*’s<0.003).

When heat thresholds were set at the 80th percentile (Tmean = 18.2 °C, Tmax = 24.6 °C, and Tmin = 13.2 °C), none of the mean differences in log-transformed outcomes were significant except when increasing daily overall temperatures by 3 degrees which resulted in an increase in the MB-CDI score (θ = 0.08, *P* < 0.001; Supplementary Figure 11; https://links.lww.com/EE/A374). When heat thresholds were set at the 85th percentiles (Tmean = 19.3 °C, Tmax = 25.9 °C, and Tmin = 14.1 °C), none of the mean differences in log-transformed outcomes were significant when increasing daily temperatures by 1 degree. Increasing daily temperatures by 2 degrees resulted in decreased MB-CDI scores for overall temperature (θ = −0.24, *P* = 0.04; Supplementary Figure 12; https://links.lww.com/EE/A374), but increased MB-CDI scores for nighttime temperatures (θ = 0.14, *P* = 0.05). Finally, increasing overall and nighttime temperatures by three degrees resulted in decreased MB-CDI scores by at least 15% (θ’s<−0.15, *P*’s < 0.001).

## Discussion

In this study, we applied the LMTP framework to evaluate the impact of temperature increases during prenatal and postnatal periods on linguistic development in 2-year-old children. Our study demonstrates a negative effect of air temperature increases on the MB-CDI score across multiple analyses in counterfactual scenarios where temperature increases by two and three degrees. Our findings indicate stronger and more consistent associations between heat events and language impairments when heat events were defined using the 90th percentile temperature threshold. In contrast, definitions based on the 85th and 80th percentiles yielded less consistent associations, including two unexpected positive associations (where increased heat events were linked to higher MB-CDI scores). This may be because lower percentile thresholds capture less intense heat exposure that is unlikely to induce physiological heat stress (at the 90th percentile, Tmean = 20.6 °C; at the 85th percentile, Tmean = 19.3 °C; and at the 80th percentile, Tmean = 18.2 °C). Lower percentile thresholds may thereby have introduced noise into heat exposure measurement.

Our findings align with the biological plausibility of the relationship between heat exposure and neurodevelopment,^38^ and with our previous analysis of the same dataset using standard distributed lag non-linear modeling (DLNM) and static counterfactual comparisons.^16^ Compared to our previous analysis, however, the LMTP approach has direct policy relevance, as it can be used to model shifts in current exposures. For example, our analysis indicates that a three-degree increase in daily temperatures, a scenario deemed plausible by the IPCC, could significantly impair linguistic development. This finding is likely to be of greater practical interest to policymakers than results based on static scenarios (as in our previous study). As discussed above, such static exposures may not reflect real-world conditions, which could lead to the findings being underestimated or even discounted, thereby downplaying the potential consequences of climate change on early childhood development.

In addition to enabling more realistic modeling of temperature exposure effects, LMTP offers three particularly noteworthy advantages that reduce threats to the identifiability of causal effects, limit bias, and improve the precision in causal estimates. First, aside from the assumption of no unmeasured confounding, the identification of causal effects depends on the assumption that all individuals could, in principle, experience any level of exposure at each time point throughout the study period.^36^ In practice, however, individuals with certain characteristics may never be exposed to static temperatures at particular times. Previous studies have often either overlooked such potential positivity violations and relied on extrapolation to estimate exposure effects in scenarios that are rare or implausible for some participants, or they have restricted analyses to subpopulations where the exposures of interest actually occur, which may introduce selection bias and limit generalizability. Another strategy is to account for regional acclimatization by defining region-specific reference exposures, for example, through multiple regional sub-analyses synthesized in a meta-analysis.^39^ Although individuals are more likely to experience temperature extremes relative to their local climate than to global averages, this approach does not ensure that all exposures are plausible for every participant at every time point across the study period. By contrast, LMTP leverages changes in exposure based on each individual’s observed exposures, thereby increasing the plausibility that all individuals can be exposed to counterfactual scenarios throughout the study period, that is, decreasing the likelihood of positivity violations.

Second, standard practices in environmental epidemiology can accommodate nonlinear effects and interactions, provided these relationships are specified a priori based on theoretical assumptions or prior knowledge. However, this approach may not capture unexpected or complex patterns present in the data. Furthermore, previous studies have not explicitly accounted for the probability of exposures given the covariates, which is important for addressing potential selection bias and achieving covariate balance across exposure levels. Even if the appropriate set of confounders is included in the model, failing to systematically account for complex interactions between covariates and both the outcome and the exposure can result in residual confounding.^40^ Additionally, inadequate covariate balance across exposure groups can compromise comparability between participants.^41^ Longitudinal settings introduce further complexity, with time-dependent confounders both influencing further exposures and being influenced by previous exposures, while also affecting the outcome. Traditional regression approaches cannot capture this intricate web of temporally ordered relationships, and while DLNM can address distributed or delayed effects of exposures over time, it does not inherently account for such broader complexities in causal structures. By contrast, LMTP leverages ML to model both the outcome regression and the exposure mechanism and is specifically designed to accommodate complex interrelationships between time-varying exposures and confounders.

Third, standard regression models often fail to specifically target the relationship between the exposure and the outcome, as they generally treat the exposure variable as just another covariate. While this approach yields coefficients and precision measures that are valid when all predictors are of equal interest, it is less suitable for causal inference, where the primary goal is to accurately estimate the exposure-response relationship. By integrating TMLE, LMTP attains a bias-variance tradeoff tailored to the causal parameter of interest. This is accomplished by robustly estimating both the outcome regression and the exposure mechanisms (with the aid of ML techniques), and then updating the outcome regression estimates based on information from the exposure mechanism.^42^ By employing such doubly robust estimation techniques, LMTP also solves the efficient influence function estimating equation, enabling valid statistical inference.^33^

### Limitations

First, residual confounding remains a possibility due to potential omission or misspecification of confounders, especially time-dependent confounders (e.g., vegetation or household income). Another potential source of bias arises from the fact that, for some observations, language production was reported by fathers rather than mothers. Mothers may provide more accurate assessments than fathers; however, this applied to only 104 participants, so any resulting bias is likely to be minimal. Second, the results of our sensitivity analyses, which used lower heat thresholds to define heat spikes, did not fully replicate our primary findings. Lowering the threshold to the 80th percentile may be overly conservative in the context of France’s relatively cool 2010–2012 climate. At the 85th percentile, CIs for counterfactual mean differences almost overlapped with zero when simulating a 2 °C temperature increase, introducing uncertainty in the results. However, results under a 3 °C warming scenario showed greater alignment with our primary findings, suggesting threshold-specific sensitivity in the observed effects. Third, some of the density ratios were relatively high over the study period. High density ratios indicate limited overlap between exposure groups, which can compromise the validity of causal estimates by increasing reliance on a small subset of the data. As density ratios increase, so does the variance of the estimator, resulting in wider CIs and less precise estimates. To mitigate this issue, we truncated the density ratios at the 99.9th percentile. Fourth, unlike DLNM, LMTP is not designed to identify specific sensitive windows of exposure. We view these methods as complementary, with LMTP providing robust causal estimates for overall exposure effects and DLNM offering insights into temporal patterns of vulnerability. Fifth, we also acknowledge that truncating exposure data after 21 months may have been overly conservative and could have excluded effects occurring closer to the time of outcome assessment. Future studies should explore whether a latency exists between heat-related biomolecular injury and subsequent language impairments in humans.

Sixth, the LMTP framework enables the utilization of interesting features, which we have not used in the current study. For instance, counterfactual exposures could have been drawn stochastically, for example, from a Gaussian distribution of mean {1,2,3} °C and standard deviation {0.5, 2} °C, instead of being set up deterministically.^26^ Likewise, the shift function could have been based on the region of the participant, as it has been suggested that those exposed to a Mediterranean climate may be subject to a higher temperature increase than those living in the north of France.^43^ Finally, new applications investigating causal mediation in the context of LMTP are being tested.^44^ Applied to our case, this may translate into a research question testing whether increases in temperature directly affect linguistic development vs. indirectly via changes in air pollutants’ concentrations.

Seventh, despite the large sample size, only 51% of eligible mothers consented to participate, raising the question of the representativeness of the population. However, as explained by Rothman et al.^45^, “representativeness does not, in and of itself, deliver valid scientific inference.” Additionally, among participating mothers, missing values raise the potential risk of selection bias.

## Conclusions

Our study demonstrates the application of LMTP to assess the impact of air temperature changes on linguistic development in 2-year-old children. The LMTP framework offers several advantages: it enables testing of realistic, data-driven counterfactual exposures, relaxing the positivity assumption; accommodates complex data structures to model accurate relationships between variables; and employs doubly robust estimation techniques. These features enhance the identifiability of causal estimates and the statistical robustness of causal parameter estimation.

Applying the LMTP framework, we found that a three-degree increase in overall, daytime, and nighttime temperature exposures during early life consistently reduced linguistic abilities in children. We hope that this study will serve as a catalyst for increased adoption of the LMTP approach in environmental research.

## Conflicts of interest statement

The authors declare that they have no conflicts of interest with regard to the content of this report.

## Acknowledgments

The Elfe survey is a joint project between the French Institute for Demographic Studies (INED) and the National Institute of Health and Medical Research (INSERM), in partnership with the French blood transfusion service (Etablissement français du sang, EFS), Santé publique France, the National Institute for Statistics and Economic Studies (INSEE), the Direction générale de la santé (DGS, part of the Ministry of Health and Social Affairs), the Direction générale de la prévention des risques (DGPR, Ministry for the Environment), the Direction de la recherche, des études, de l’évaluation et des statistiques (DREES, Ministry of Health and Social Affairs), the Département des études, de la prospective et des statistiques (DEPS, Ministry of Culture), and the Caisse nationale des allocations familiales (CNAF), with the support of the Ministry of Higher Education and Research and the Institut national de la jeunesse et de l’éducation populaire (INJEP). Via the RECONAI platform, it receives a government grant managed by the National Research Agency under the “Investissements d’avenir” programme (ANR-11-EQPX-0038 and ANR19COHO-0001).

## Supplementary Material
