# Supplementary material for: Effects of projected increases in heat exposure on linguistic development in two-year-old children: A longitudinal modified treatment policy analysis
Source: Environ Epidemiol. 2025 Oct 13;9(6):e423. doi: 10.1097/EE9.0000000000000423 (PMC12520208; doi:10.1097/EE9.0000000000000423)
Supplement: Supplementary file 1 [file ee9-9-e423-s001.pdf]

Effects of projected increases in heat exposure on linguistic development in two-year-old children: a longitudinal modified treatment policy analysis

Table of Contents

Supplementary Method. Description of the covariates. 2
Supplementary Table. Socio-demographic characteristics of the study population. 5
Supplementary Figure 1. Counterfactual distributions of daily temperatures....9
Supplementary Figure 2. Counterfactual heat exposure distributions based on overall temperature (Tmean)....10
Supplementary Figure 3. Counterfactual heat exposure distributions based on daytime temperature (Tmax)....11
Supplementary Figure 4. Counterfactual heat exposure distributions based on night-time temperature (Tmin)...12
Supplementary Figure 5. Counterfactual heat exposures based on overall temperature (Tmean) with heat thresholds set at the 80th and 85th percentiles....13
Supplementary Figure 6. Counterfactual heat exposures based on daytime temperature (Tmax) with heat thresholds set at the 80th and 85th percentiles....14
Supplementary Figure 7. Counterfactual heat exposures based on night-time temperature (Tmin) with heat thresholds set at the 80th and 85th percentiles....15
Supplementary Figure 8. Boxplots of estimated density ratios for counterfactual scenarios based on overall temperatures (Tmean)....16
Supplementary Figure 9. Boxplots of estimated density ratios for counterfactual scenarios based on daytime temperature (Tmax)....17
Supplementary Figure 10. Boxplots of estimated density ratios for counterfactual scenarios based on night-time temperature (Tmin)....18
Supplementary Figure 11. Effects of rising daily air temperatures on MB-CDI scores with heat thresholds set at the 80th percentile....19
Supplementary Figure 12. Effects of rising daily air temperatures on MB-CDI scores with heat thresholds set at the 85th percentile....20
References....21

Supplementary Method. Description of the covariates.

- Parents: socio-economic status and demographic indicators.

Numerous studies have demonstrated that a high socio-economic status – characterized by factors such as high household income, maternal education, and professional occupation – can create a supportive environment for language development [1]. Parental age at conception has also been related to a child's cognitive abilities: on the one hand, high maternal [2] and paternal age [3,4] at conception have been related to a range of adverse perinatal outcomes. Yet on the other hand, advanced maternal age has been related to a socioeconomically advantaged family background [5]. Children also exhibit lower performances in language abilities when their parents are separated [6], though this finding is controversial [7]. Finally, others have reported cognitive development disparities based on racial or ethnic origins [8].

Consequently, we adjusted our analyses on parental education, measured when the child was two months of age (highest level among mother and father: primary or intermediary school vs. high-school vs. undergraduate diploma (Baccalaureate +two years) vs. graduate diploma (Baccalaureate +two to five years) vs. postgraduate diploma (Baccalaureate +five years or more)); maternal and paternal age at conception (categorical – five year bins); socio-economic position of the mother, measured in the second trimester (executives and higher intellectual professions vs. intermediate professions vs. employees vs. workers vs. craftsmen or merchants vs. others); household income, measured when the child was two years of age (linearly ordered quintiles); and whether parents were living together when the child was two years of age (no vs. yes). Though we did not record race and ethnicity because racial statistics are not allowed in France [9], we adjusted our analysis on the mother's birthplace (France vs. other).

- Neighborhood: socio-economic context and urbanization.

Both socially deprived and urbanized areas may show higher levels of temperature [10,11]. On the other hand, a child's neighbourhood, and especially its socio-cultural context, may influence the quality of his/her home learning environment [12], and in turn decrease his/her learning abilities.

We adjusted our analyses for an index of urbanization, measured when the child was one year of age, with three different categories based on the size of the urban area (not an urban area or urban area with less than 50,000 inhabitants vs. urban area with 50,000 to 500,000 inhabitants vs. urban area with more than 500,000 inhabitants). We also adjusted for the European Deprivation Index obtained for the year 2011, at the smallest geographical unit available in France, which is the census block group level, known as IRIS in Metropolitan France (EDI [13]). The EDI was categorized in three different groups based on tertiles to account for non-linear effects.

- Neighbourhood: vegetation.

Vegetation can affect thermal conditions [14] and cognitive development [15,16]. We adjusted for the normalized difference vegetation index (NDVI) as the mean Landsat satellite NDVI in a 100 m buffer around home address during the months of June, July and August of years 2011, 2012, 2013. Values calculated with less than 75% observations were removed and considered missing. NDVI was further categorized in three different groups based on tertiles to account for non-linear effects.

- Languages spoken at home.

Language exposure has been shown to have an effect on language development [17]. The number of languages spoken at home, measured when the child was two years of age, was included as a covariate in our analysis (one vs. two vs. three or more).

- Pre-pregnancy history: parity.

It has been argued that first-born children have better cognitive outcomes than later-born children, potentially because they have greater access to parental resources [18]. Also, there may be a negative relationship between the size of the family and the level of household income [5,19], which may explain why large family size is negatively associated with early cognitive development [20]. Parity (primiparous vs. multiparous) was included as a covariate.

- Pre-pregnancy history: neurodevelopmental difficulties.

A parental history of neurodevelopmental issues may predispose to neurodevelopmental disorders in the offspring. Consequently, we adjusted our analysis for mother's and father's history of problems with mathematics, reading, writing, communication and language (at least one issue vs. no impairment).

- Pre-pregnancy history: maternal obesity.
Maternal obesity has been shown to be associated with neurodevelopment of two-year-old children [21]. We therefore adjusted for maternal Body Mass Index (BMI) before pregnancy (BMI<18.5 vs. BMI between 18.5 and 25 vs. BMI between 25 and 30 vs. BMI>30).

- Food and drug exposure during pregnancy and after birth.
Maternal supplementation in vitamin B9 is thought to facilitate brain development [22]. Smoking during pregnancy has been suggested to reduce speech processing abilities [23], see however contradictory findings [24], and [25] for review). Studies have demonstrated a negative effect of alcohol consumption during pregnancy on children's language abilities [26]. Caffeine exposure has been negatively related to children's full-scale and performance IQ (but not verbal IQ) compared with low caffeine exposure [27]. Yet, no significant associations were found between caffeine intake during pregnancy and language difficulties at the age of 18 months [28]. Maternal fish consumption has been associated with a child's language abilities at age three [29,30]. Fish is thought to be beneficial to the fetus via their content in poly-unsaturated fatty acid (a.k.a. omega 3). For instance, a recent study has found that mothers consuming omega-3 in the second half of pregnancy were 40% less likely to have children with autism spectrum disorders [31]. Breastfeeding has been associated with a range of positive neurodevelopmental outcomes [32].

We adjusted our analysis for maternal supplementation in vitamin B9, measured in the third trimester (no vs. yes); smoking during pregnancy, measured in the second trimester (not exposed vs. actively or passively exposed); alcohol use during pregnancy, measured in the second trimester (less than once per month vs. more than once per month); coffee consumption during pregnancy, measured in the third trimester (less than once a day vs. more than once a day); fish consumption during pregnancy, measured in the second trimester (never vs. less than once a month vs. once to three times a month vs. once a week vs. more than once a week; linearly ordered); fatty acid supplementation, measured in the third trimester (no vs. yes); and breastfeeding, measured when the child was two months of age (breastfeeding only vs. breastfeeding and bottle feeding vs. bottle feeding only).

- Age and sex of the child.
Boys have been shown to be a bit delayed in their language development [33]. We included the sex of the child (male vs. female) as a covariate in the analysis and subsequently stratified our analysis by sex (see below). We also included the child's exact age (in months) at the time the MB-CDI was scored as a covariate.

- Air pollution.
Ambient temperature has been suggested to influence air pollution concentration via its effect on emissions, atmospheric chemistry and pollutant transport [34,35]. Air pollution may itself impact cognitive development [36]. In our analysis, we investigated the total effect of temperature on language development, not accounting for pollution.

- Pregnancy, birth issues and medical history of the child.
Though potentially involved in neurodevelopment, we did not adjust for pregnancy and birth outcomes, as well as medical issues during infancy. Indeed, the latter may be caused by ambient temperatures that are above or below average and may be considered mediators.

- Factors that may be influenced by linguistic development.
External stimulation (whether from parents, other adults or children), childcare and preschool attendance, can influence children's ability to speak – e.g. via language input [37]. Association between screen use and language development has also proven significant, with greater quantity of screen use being negatively associated with child language [38]. Sleep is another feature typically involved in language development [39].

The reverse however may also be true. Preschool children with language impairment have been shown to suffer from emotional (e.g. understanding emotion meaning [40]) and behavioral problems (e.g. hyperactivity [41]), as well as poor social competence [42], and have been found to be more dependent and isolated [43]. The latter in

turn may influence the quality and quantity of external stimulations, childcare or preschool attendance, and quality of sleep [44,45].

In addition, evidence of bidirectional associations between sensitive parenting and language skills has been observed, especially in boys. For instance, boys' receptive language skills at 24 months uniquely contributed to increased sensitive parenting by mothers from 24 to 36 months [46]. Another study reported evidence of child-to-parent effects, where children with higher language skills tend to elicit more complex language input from their parents [47].

Accordingly, we reasoned that language development may influence stimulation and activities, childcare, preschool attendance, screen use and sleep, and that adjusting our analysis for these factors would risk reverse causation bias.

- Time-dependent confounders

Cold spells in each 10-week period were used as time-dependent confounders, as in Wang et al [48]. Cold spikes can influence the likelihood or intensity of future heat spikes due to seasonal patterns and autocorrelation in temperature data. In addition, populations accustomed to cold may become less physiologically or behaviorally prepared for heat spikes, due to potential genetic trade-offs [49] and a lack of heat-specific adaptations. Finally, cold exposure has been shown to influence neurodevelopment [50].

Similarly to heat, cold was defined as the number of times at least two consecutive days had daily temperatures below a cold threshold in each period. Cold thresholds were defined as the 20th, 15th and 10th percentiles where heat thresholds were defined as the 80th, 85th and 90th percentiles, respectively.

Supplementary Table. Socio-demographic characteristics of the study population.

Variables	Total (N=12163)
**MB-CDI Score**	
Median [Q1, Q3]	81 [59, 93]
**Daily overall temperature[a] (Tmean, °C)**	
Mean [min, max]	12.1 [-20.1, 31.8]
**Daily daytime temperature[a] (Tmax, °C)**	
Mean [min, max]	17.3 [-15.7, 42.8]
**Daily night-time temperature[a] (Tmin, °C)**	
Mean [min, max]	7.7 [-24.3, 29.1]
**Daily PM2.5 concentration[a] (μg/m³)**	
Mean [min, max]	15.3 [0, 108]
**Daily PM10 concentration[a] (μg/m³)**	
Mean [min, max]	22.5 [0, 122]
**Daily NO2 concentration[a] (μg/m³)**	
Mean [min, max]	20.2 [0, 238]
**European Defavor Index of living area**	
Low: (-9.18,-1.6]	4434 (36.5%)
Medium: (-1.6,2.06]	4194 (34.5%)
High: (2.06,31.9]	3519 (28.9%)
Missing	16 (0.1%)
**NDVI**	
Low: (0.0367,0.393]	3732 (30.7%)
Medium: (0.393,0.51]	4066 (33.4%)
High: (0.51,0.89]	4362 (35.9%)
Missing	3 (0.0%)
**Size of living area**	
Rural area or less than 50,000 inhabitants	2447 (20.1%)
50,000 to 500,000 inhabitants	3778 (31.1%)
More than 500,000 inhabitants	5543 (45.6%)
Missing	395 (3.2%)
**Mother's birth place**	
France	10903 (89.6%)
Overseas	1185 (9.7%)
Missing	75 (0.6%)
**Mother's history of learning difficulties**	
No	6715 (55.2%)

Variables	Total<br>(N=12163)
Yes	4978 (40.9%)
Missing	470 (3.9%)
**Father's history of learning difficulties**	
No	4964 (40.8%)
Yes	5017 (41.2%)
Missing	2182 (17.9%)
**Level of education (Highest between mother and father)**	
Primary or secondary school	264 (2.2%)
Highschool	2931 (24.1%)
Undergraduate	2675 (22.0%)
Bachelor	2191 (18.0%)
Postgraduate	3914 (32.2%)
Missing	188 (1.5%)
**Mother's socio-professional category**	
Craftsmen & merchants	1010 (8.3%)
Executives and higher intellectual professions	3011 (24.8%)
Intermediate professions	1739 (14.3%)
Employees (skilled and unskilled)	4234 (34.8%)
Workers (skilled and unskilled)	1377 (11.3%)
Others	532 (4.4%)
Missing	260 (2.1%)
**Parity**	
Primiparous	5531 (45.5%)
Multiparous	6586 (54.1%)
Missing	46 (0.4%)
**Pre-pregnancy Body Mass Index**	
<18.5	868 (7.1%)
18.5-25	8053 (66.2%)
25-30	2010 (16.5%)
>30	1120 (9.2%)
Missing	112 (0.9%)
**Mother's age at conception**	
25 or below	1139 (9.4%)
26-30	3988 (32.8%)
31-35	4521 (37.2%)
36-40	2046 (16.8%)

Variables	Total (N=12163)
41 or above	453 (3.7%)
Missing	16 (0.1%)
**Father's age at conception**	
25 or below	554 (4.6%)
26-30	2730 (22.4%)
31-35	4332 (35.6%)
36-40	2693 (22.1%)
41-45	1048 (8.6%)
46 or above	463 (3.8%)
Missing	343 (2.8%)
**Alcohol consumption during pregnancy**	
At least once a month	3128 (25.7%)
Never or less than once a month	8955 (73.6%)
Missing	80 (0.7%)
**Consumption of tobacco during pregnancy**	
At least some exposure (passive or active)	3844 (31.6%)
No exposure	7968 (65.5%)
Missing	351 (2.9%)
**Coffee consumption during pregnancy**	
Less than once a day	9966 (81.9%)
Once a day or more	1030 (8.5%)
Missing	1167 (9.6%)
**Fish consumption during pregnancy**	
Never	613 (5.0%)
Less than once a month	1313 (10.8%)
One to three times a month	3294 (27.1%)
Once a week	3714 (30.5%)
Twice a week or more	2160 (17.8%)
Missing	1069 (8.8%)
**Vitamin B9 consumption during pregnancy**	
No	5250 (43.2%)
Yes	6634 (54.5%)
Missing	279 (2.3%)
**Omega 3 consumption during pregnancy**	
Less than once a week	2567 (21.1%)
More than once a week	1796 (14.8%)

Variables	Total (N=12163)
Never	5792 (47.6%)
Missing	2008 (16.5%)
**Sex of the child**	
Female	5988 (49.2%)
Male	6175 (50.8%)
**Feeding method at 2 months**	
Breastfeeding only	3963 (32.6%)
Breastfeeding & Bottle feeding	1927 (15.8%)
Bottle feeding only	5987 (49.2%)
Missing	286 (2.4%)
**Parental relationship**	
Separated	690 (5.7%)
Together	11372 (93.5%)
Missing	101 (0.8%)
**Number of languages spoken at home**	
One	8566 (70.4%)
Two	2525 (20.8%)
Three or more	834 (6.9%)
Missing	238 (2.0%)
**Household income**	
1st quintile	2168 (17.8%)
2nd quintile	2349 (19.3%)
3rd quintile	2286 (18.8%)
4th quintile	2353 (19.3%)
5th quintile	2365 (19.4%)
Missing	642 (5.3%)
**Age at MB-CDI test (month)**	
Mean [min, max]	25.33 [23.00, 28.00]
Missing	101 (0.8%)

[a] from conception to MB-CDI test
Legend. NDVI, Normalized difference vegetation index; MB-CDI, MacArthur-Bates Communicative Development Inventories; Q1, 1st quartile; Q3, 3rd quartile.

Supplementary Figure 1. Counterfactual distributions of daily temperatures

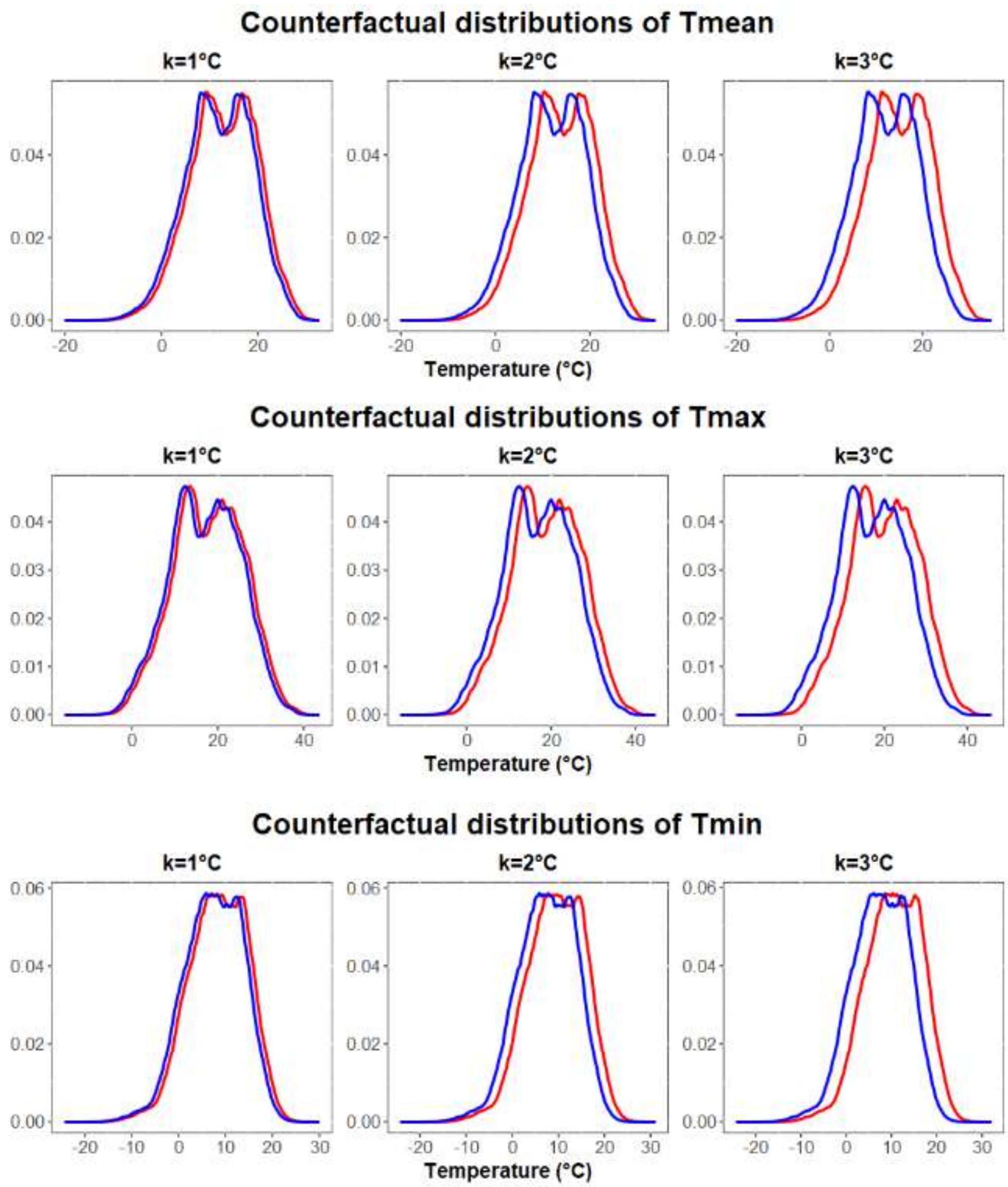

X-axis: Temperature in degree Celsius; Y-axis: Probability density.
Density plots showing the distribution of daily temperatures. Separate plots are provided for overall (mean daily temperature, Tmean), daytime (maximum temperature, Tmax), and nighttime (minimum temperature, Tmin) temperatures. Factual (blue) and counterfactual (red) exposure distributions are displayed for each counterfactual scenario (adding k=1°C, 2°C or 3°C to observed daily temperatures).

Supplementary Figure 2. Counterfactual heat exposure distributions based on overall temperature (Tmean)

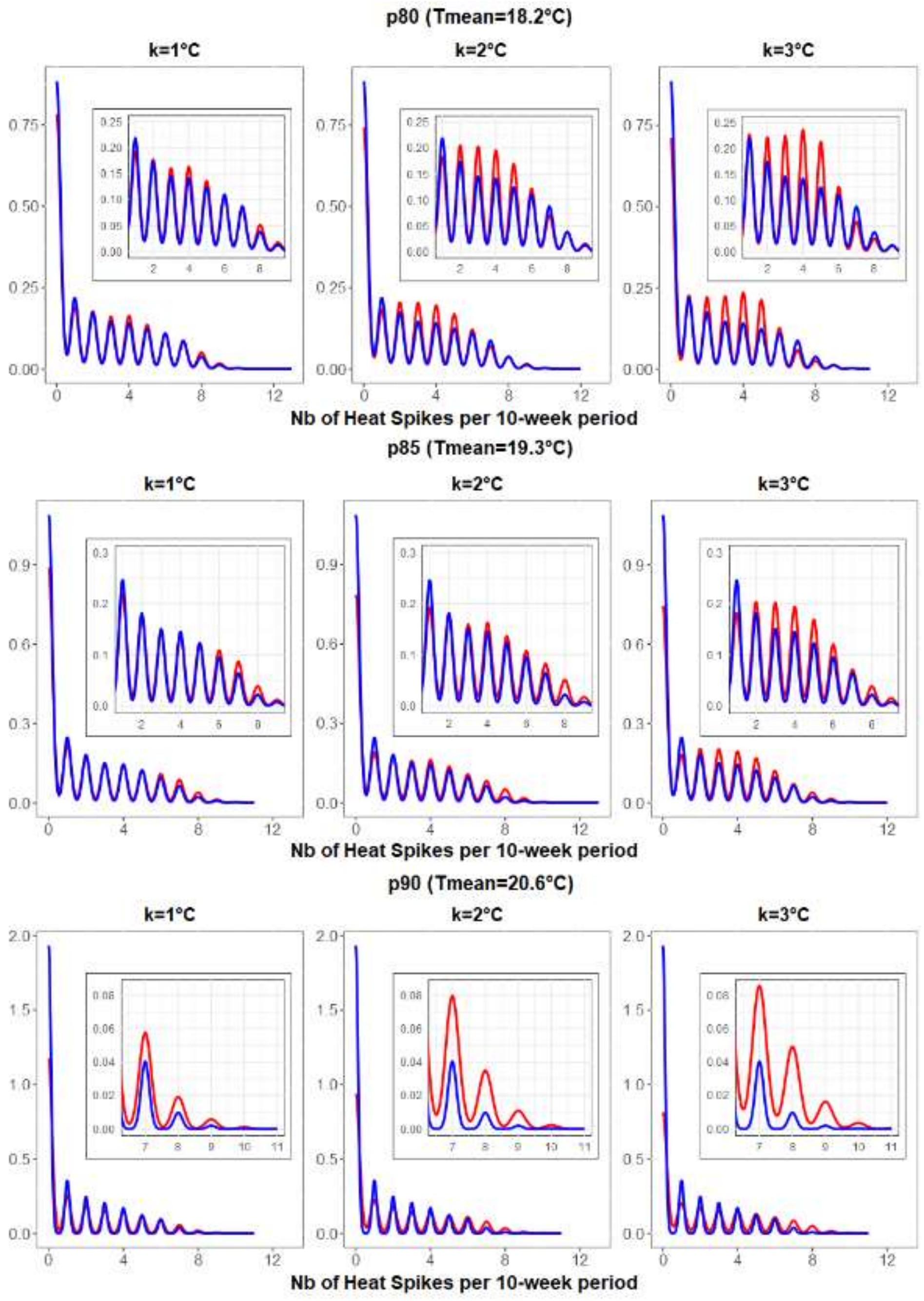

X-axis: Number of heat spikes per 10-week period; Y-axis: Probability density.
Density plots comparing factual (blue) and counterfactual (red) temperature exposure distributions.
Counterfactual scenarios represent increases of 1°C, 2°C, or 3°C added to observed daily temperatures.
Heat exposure was quantified as heat spikes, defined as the number of occurrences in each 10-week period where the daily temperature exceeded the 80th, 85th, or 90th percentiles for at least two consecutive days.
Each plot features an inset that magnifies a specific range of the data. Note the very subtle differences between the distributions.
Legend. p80, 80th percentile; p85, 85th percentile; p90, 90th percentile.

Supplementary Figure 3. Counterfactual heat exposure distributions based on daytime temperature (Tmax)

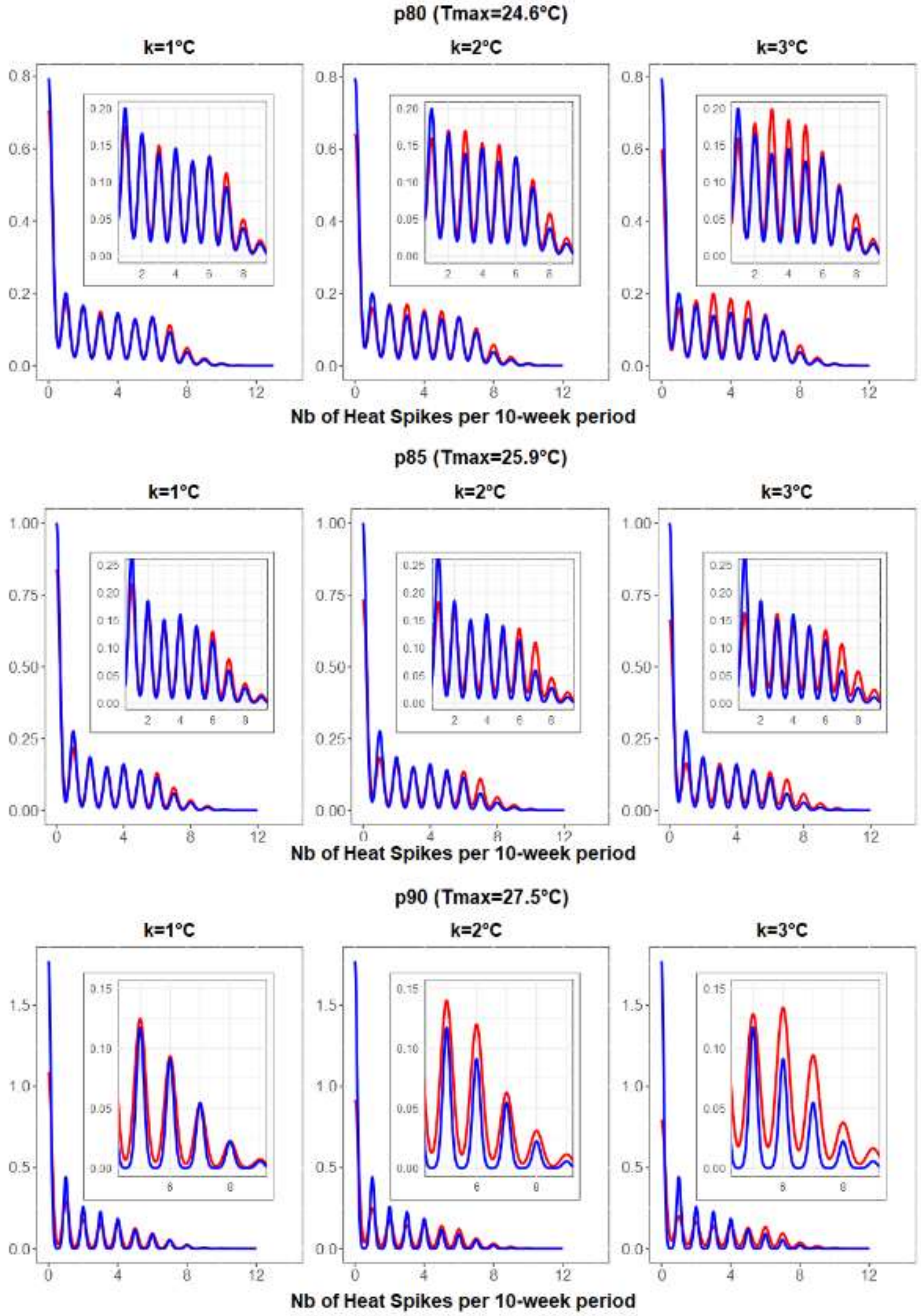

X-axis: Number of heat spikes per 10-week period; Y-axis: Probability density.
Density plots comparing factual (blue) and counterfactual (red) temperature exposure distributions. Counterfactual scenarios represent increases of 1°C, 2°C, or 3°C added to observed daily temperatures. Heat exposure was quantified as heat spikes, defined as the number of occurrences in each 10-week period where the daily temperature exceeded the 80th, 85th, or 90th percentiles for at least two consecutive days. Each plot features an inset that magnifies a specific range of the data. Note the very subtle differences between the distributions.
Legend. p80, 80th percentile; p85, 85th percentile; p90, 90th percentile.

Supplementary Figure 4. Counterfactual heat exposure distributions based on night-time temperature (Tmin)

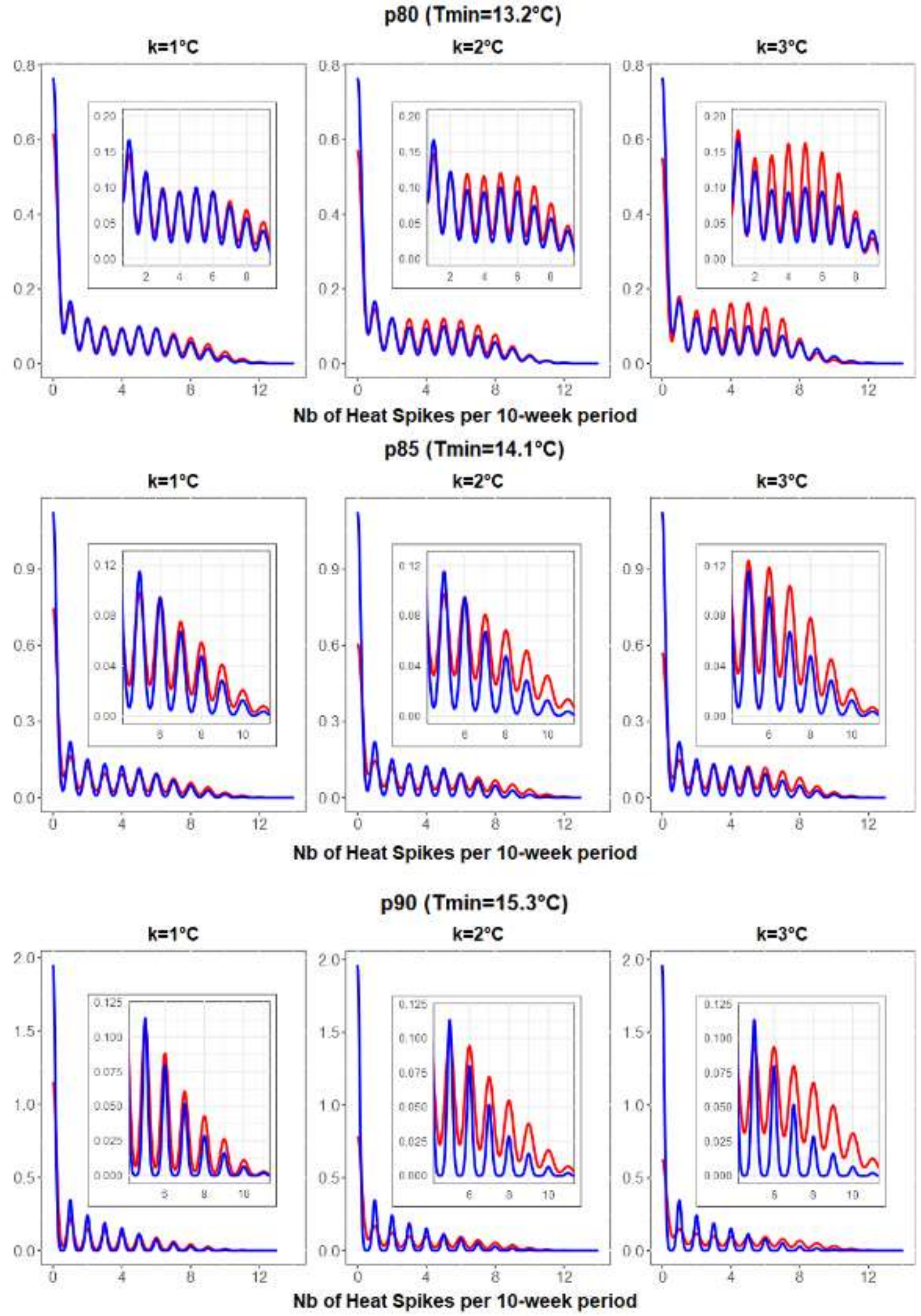

X-axis: Number of heat spikes per 10-week period; Y-axis: Probability density.
Density plots comparing factual (blue) and counterfactual (red) temperature exposure distributions.
Counterfactual scenarios represent increases of 1°C, 2°C, or 3°C added to observed daily temperatures.
Heat exposure was quantified as heat spikes, defined as the number of occurrences in each 10-week period where the daily temperature exceeded the 80th, 85th, or 90th percentiles for at least two consecutive days.
Each plot features an inset that magnifies a specific range of the data. Note the very subtle differences between the distributions.
Legend. p80, 80th percentile; p85, 85th percentile; p90, 90th percentile.

Supplementary Figure 5. Counterfactual heat exposures based on overall temperature (Tmean) with heat thresholds set at the 80th and 85th percentiles

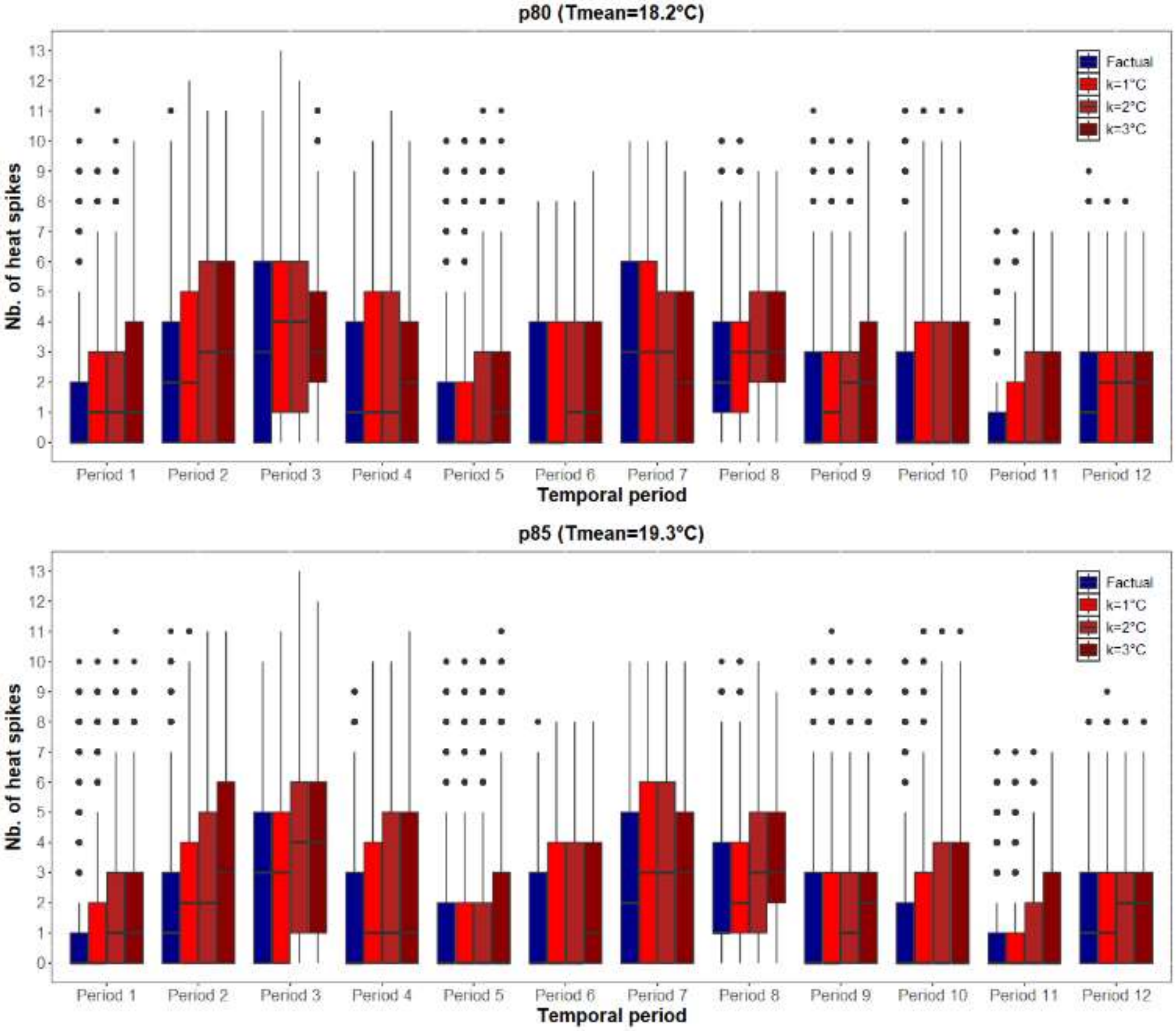

X-axis: Temporal period; Y-axis: Number of heat spikes.
Boxplots comparing factual (blue) and counterfactual (red) temperature exposures for each 10 week-period. Counterfactual scenarios represent increases of 1°C, 2°C, or 3°C added to observed daily temperatures. Heat exposure was quantified as heat spikes, defined as the number of occurrences in each 10-week period where the daily temperature exceeded the 80th and 85th percentiles for at least two consecutive days.
The box represents the interquartile range (IQR), spanning from the first quartile (Q1) to the third quartile (Q3), with the horizontal line inside the box indicating the median. The whiskers extend to the most extreme data points within 1.5 times the IQR from the quartiles, and points beyond the whiskers are plotted individually as outliers.
Legend. p80, 80th percentile; p85, 85th percentile.

Supplementary Figure 6. Counterfactual heat exposures based on daytime temperature (Tmax) with heat thresholds set at the 80th and 85th percentiles

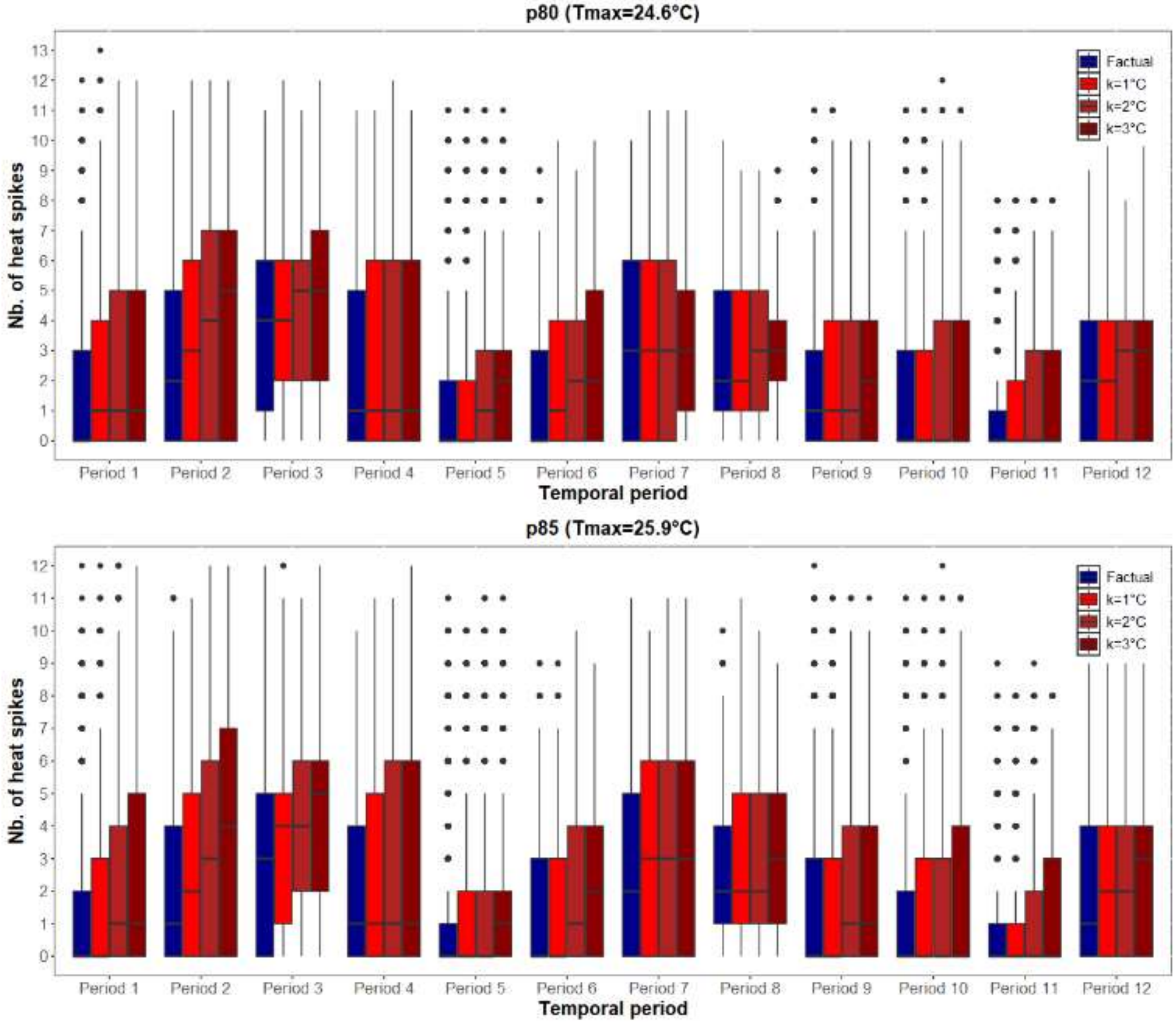

X-axis: Temporal period; Y-axis: Number of heat spikes.
Boxplots comparing factual (blue) and counterfactual (red) temperature exposures for each 10 week-period. Counterfactual scenarios represent increases of 1°C, 2°C, or 3°C added to observed daily temperatures. Heat exposure was quantified as heat spikes, defined as the number of occurrences in each 10-week period where the daily temperature exceeded the 80th and 85th percentiles for at least two consecutive days.
The box represents the interquartile range (IQR), spanning from the first quartile (Q1) to the third quartile (Q3), with the horizontal line inside the box indicating the median. The whiskers extend to the most extreme data points within 1.5 times the IQR from the quartiles, and points beyond the whiskers are plotted individually as outliers.
Legend. p80, 80th percentile; p85, 85th percentile.

Supplementary Figure 7. Counterfactual heat exposures based on night-time temperature (Tmin) with heat thresholds set at the 80th and 85th percentiles

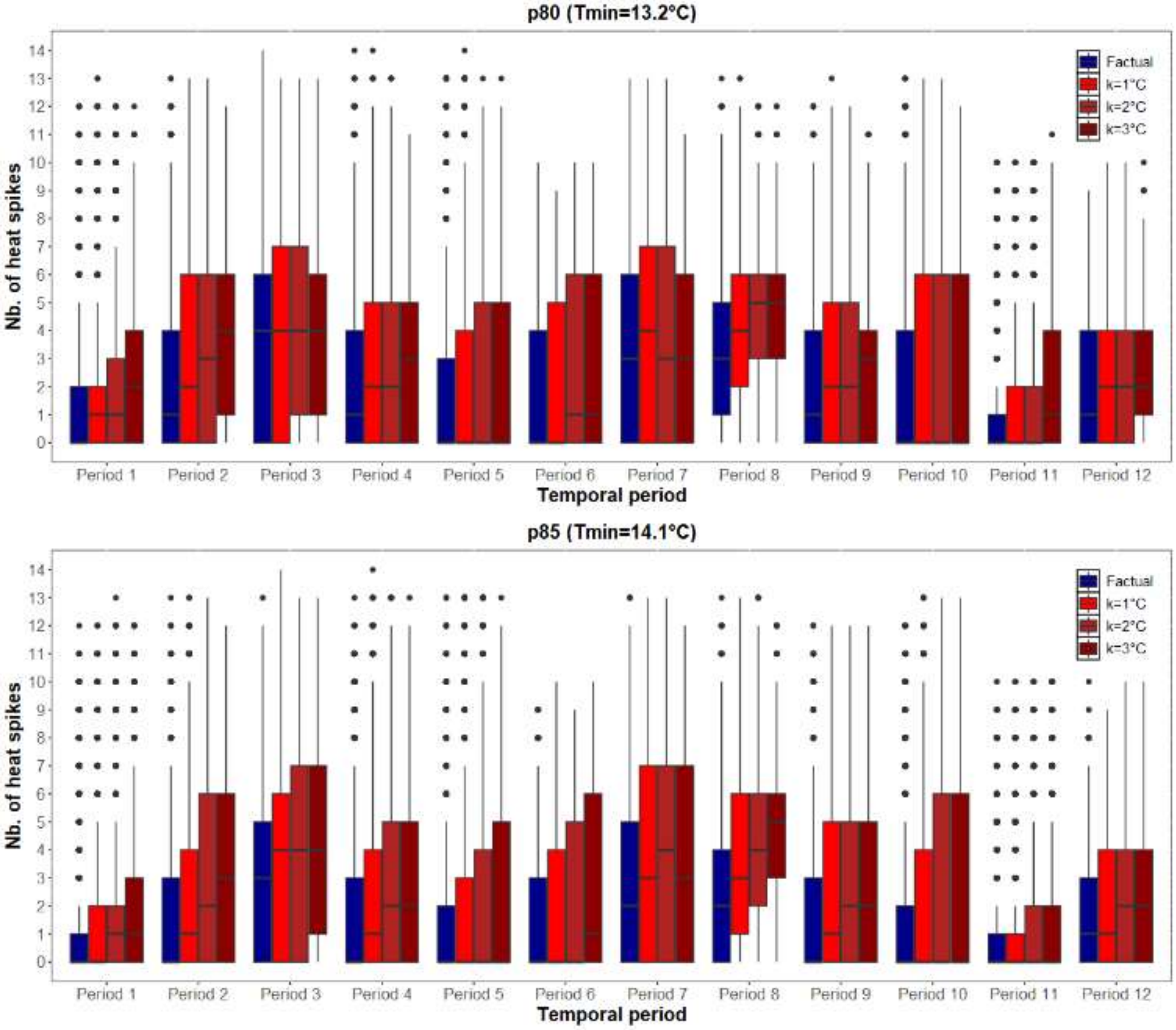

X-axis: Temporal period; Y-axis: Number of heat spikes.
Boxplots comparing factual (blue) and counterfactual (red) temperature exposures for each 10 week-period. Counterfactual scenarios represent increases of 1°C, 2°C, or 3°C added to observed daily temperatures. Heat exposure was quantified as heat spikes, defined as the number of occurrences in each 10-week period where the daily temperature exceeded the 80th and 85th percentiles for at least two consecutive days.
The box represents the interquartile range (IQR), spanning from the first quartile (Q1) to the third quartile (Q3), with the horizontal line inside the box indicating the median. The whiskers extend to the most extreme data points within 1.5 times the IQR from the quartiles, and points beyond the whiskers are plotted individually as outliers.
Legend. p80, 80th percentile; p85, 85th percentile.

Supplementary Figure 8. Boxplots of estimated density ratios for counterfactual scenarios based on overall temperatures (Tmean)

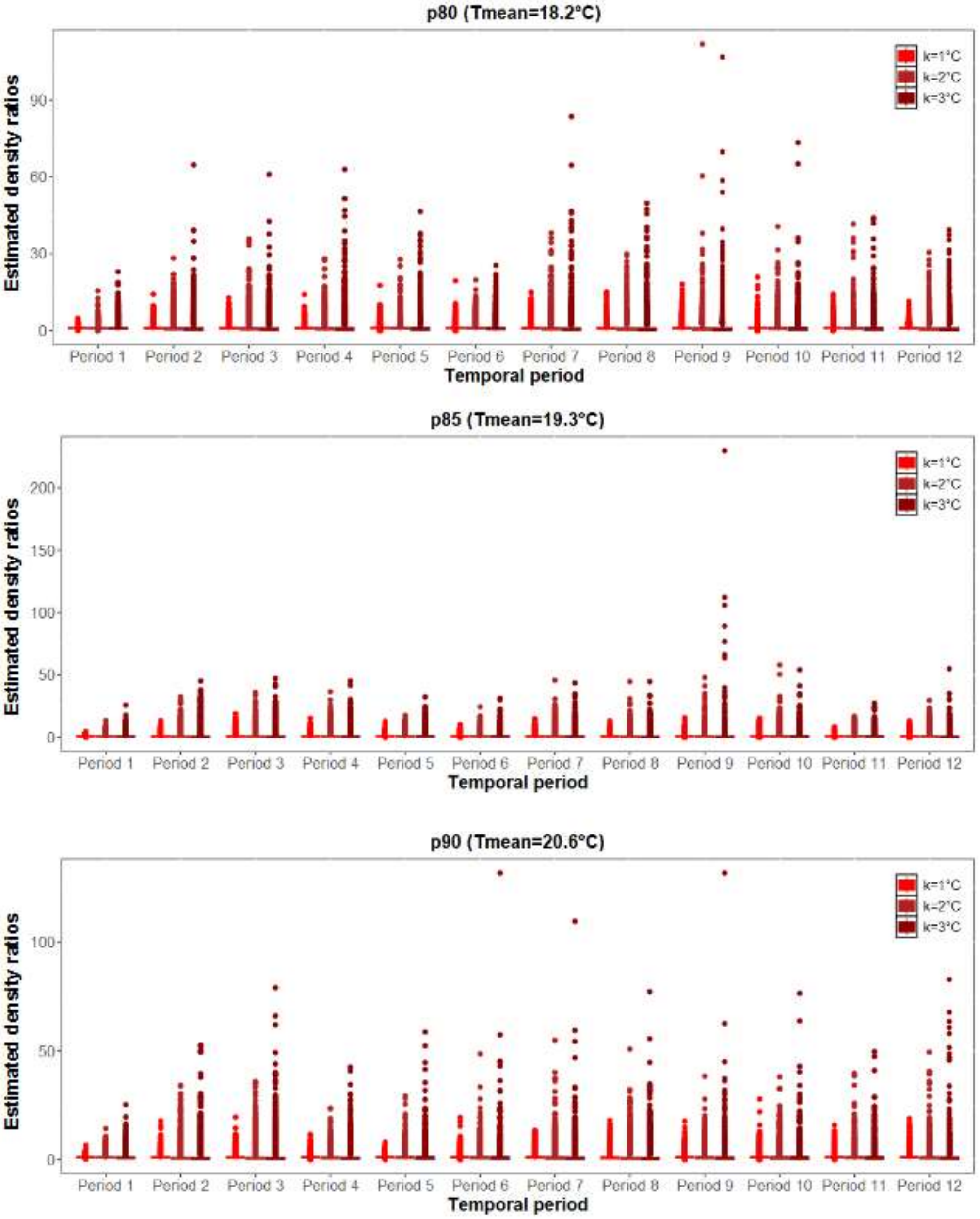

X-axis: Temporal period; Y-axis: Estimated density ratios.
Density ratios (estimates of the exposure mechanism) are plotted for each 10-week period. Counterfactual scenarios represent increases of 1°C, 2°C, or 3°C added to observed daily temperatures.
Most density ratios are below 10, although some values exceed this threshold by a substantial margin.
Legend. p80, 80th percentile; p85, 85th percentile; p90, 90th percentile.

Supplementary Figure 9. Boxplots of estimated density ratios for counterfactual scenarios based on daytime temperature (Tmax)

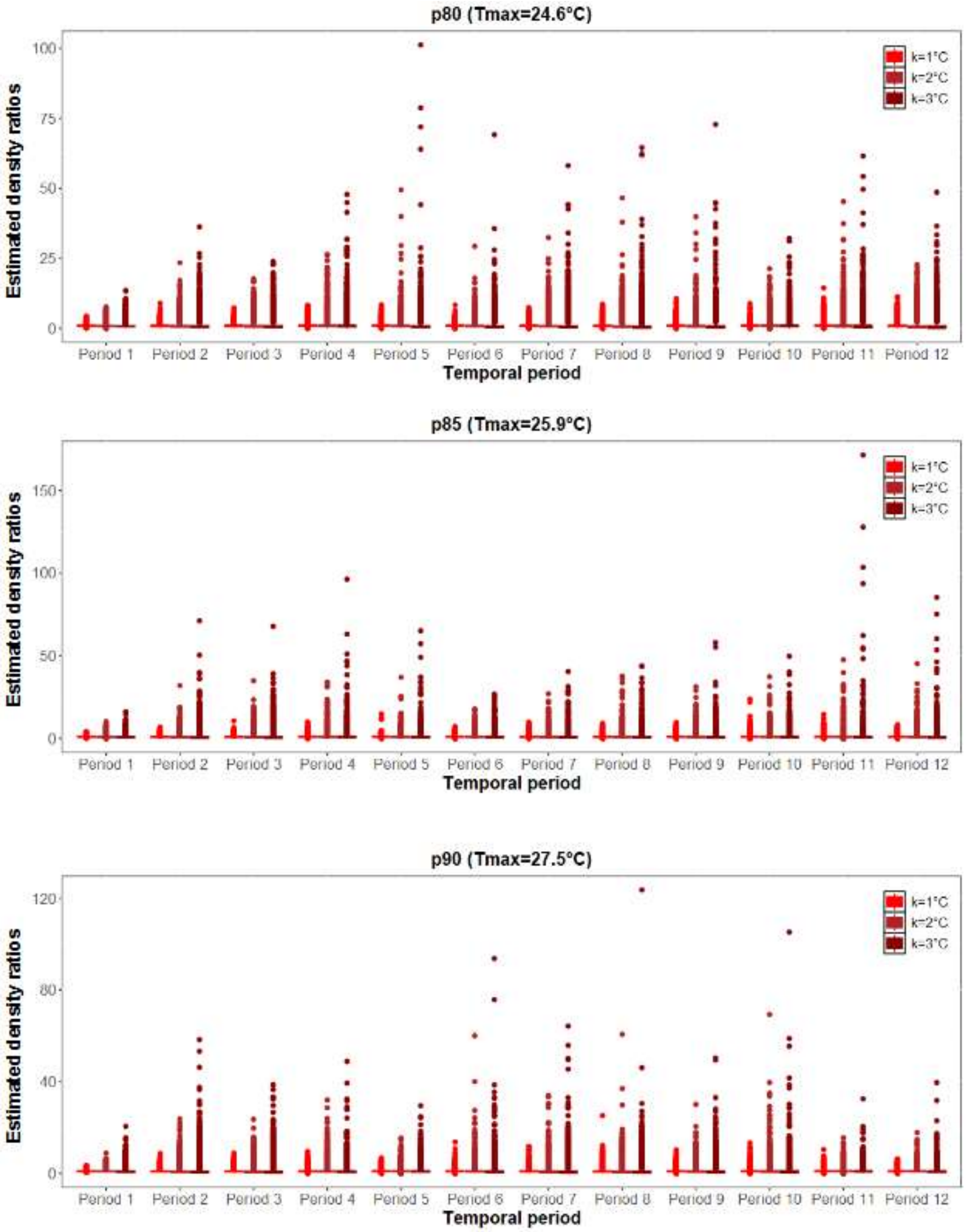

X-axis: Temporal period; Y-axis: Estimated density ratios.
Density ratios (estimates of the exposure mechanism) are plotted for each 10-week period. Counterfactual scenarios represent increases of 1°C, 2°C, or 3°C added to observed daily temperatures.
Most density ratios are below 10, although some values exceed this threshold by a substantial margin.
Legend. p80, 80th percentile; p85, 85th percentile; p90, 90th percentile.

Supplementary Figure 10. Boxplots of estimated density ratios for counterfactual scenarios based on night-time temperature (Tmin)

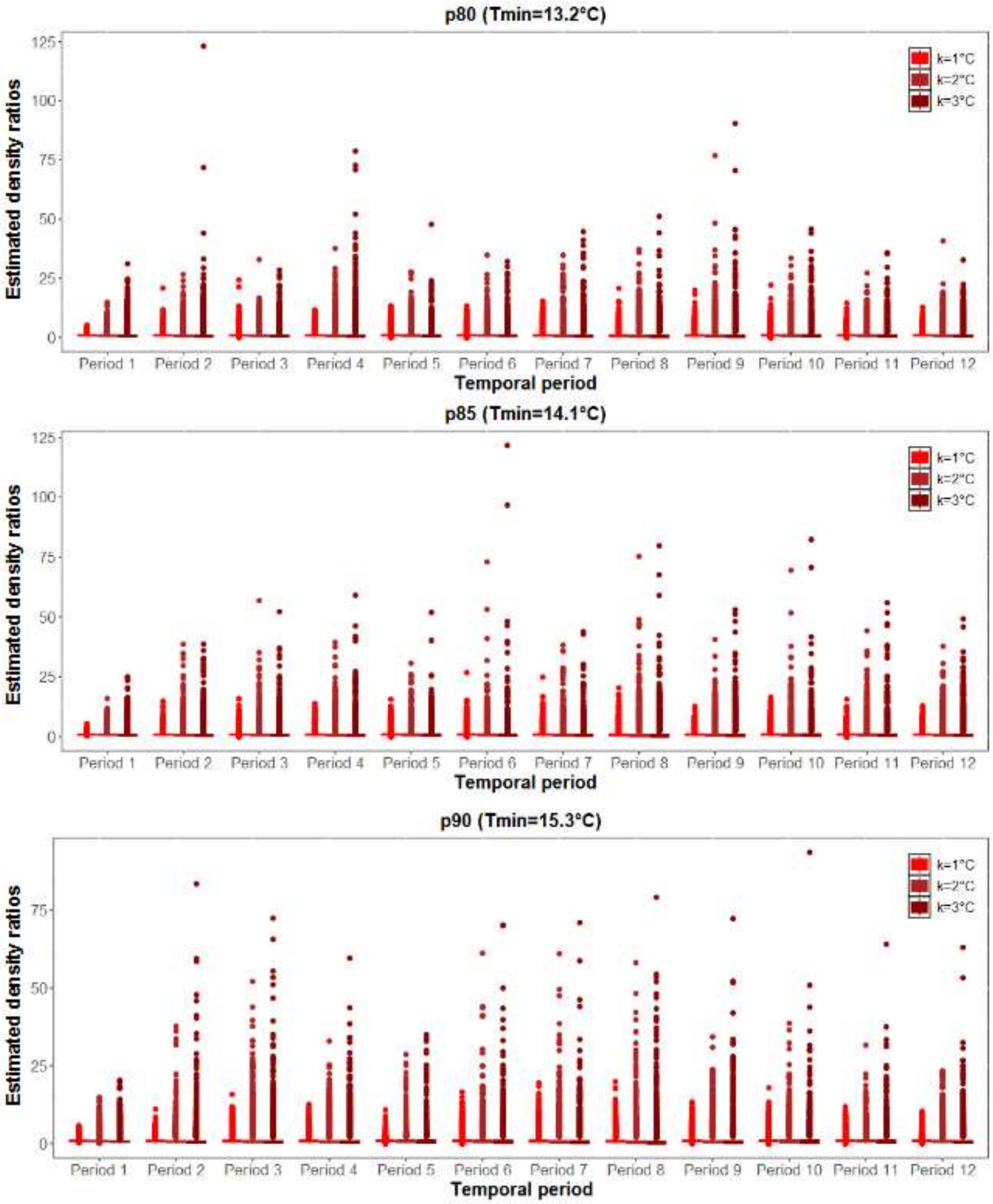

X-axis: Temporal period; Y-axis: Estimated density ratios.
Density ratios (estimates of the exposure mechanism) are plotted for each 10-week period. Counterfactual scenarios represent increases of 1°C, 2°C, or 3°C added to observed daily temperatures.
Most density ratios are below 10, although some values exceed this threshold by a substantial margin.
Legend. p80, 80th percentile; p85, 85th percentile; p90, 90th percentile.

Supplementary Figure 11. Effects of rising daily air temperatures on MB-CDI scores with heat thresholds set at the 80th percentile

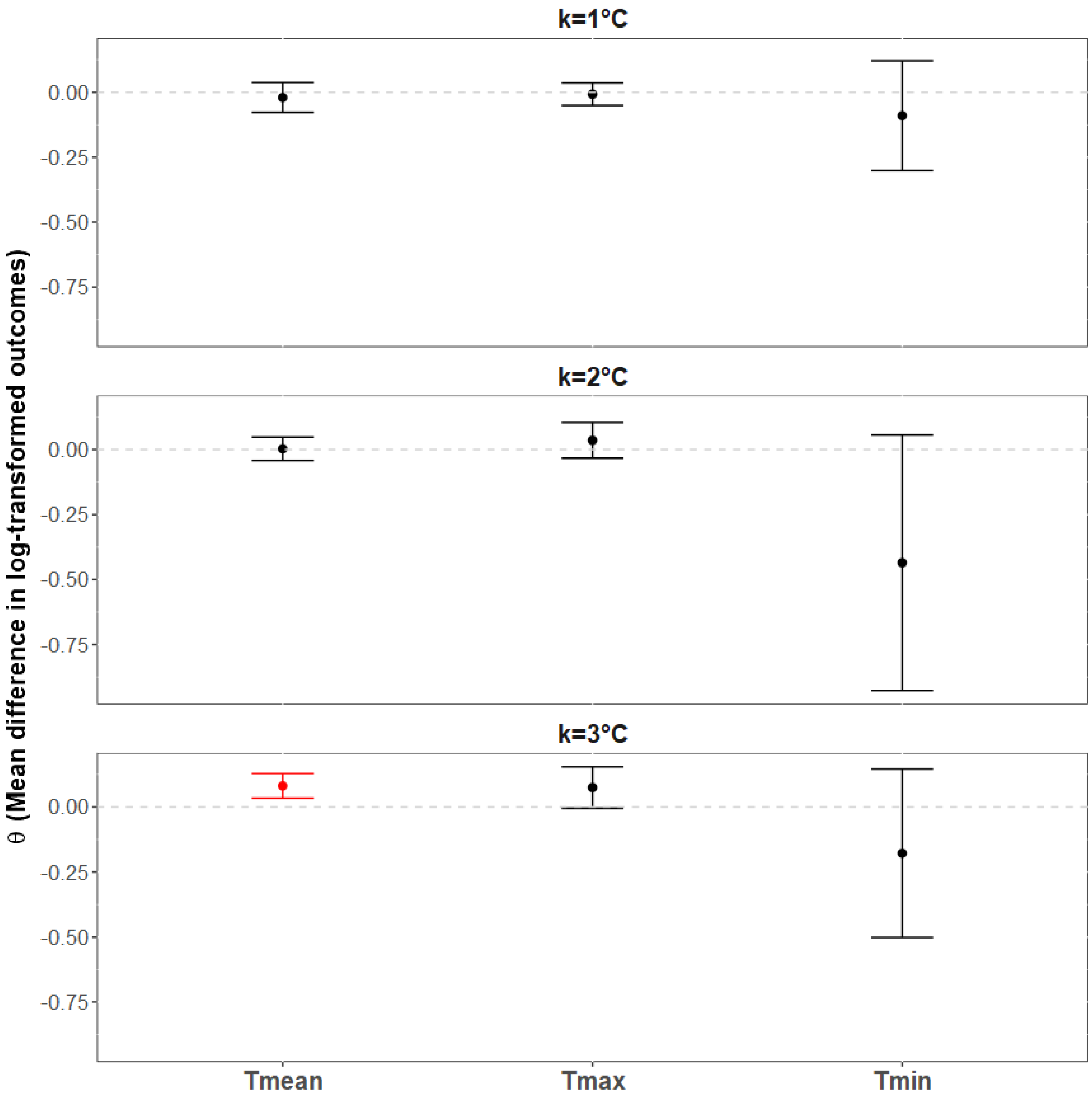

X-axis: Temperature indicators; Y-axis: Mean difference in log-transformed outcomes.
Mean differences in log-transformed MB-CDI scores are plotted along with 95% confidence intervals for counterfactual scenarios involving increases in daily temperature by 1, 2, and 3°C, with heat thresholds set at the 80th percentile for overall (Tmean=18.2°C), daytime (Tmax=24.6°C), and night-time (Tmin=13.2°C) temperatures. Estimates shown in red indicate statistical significance.
Legend. MB-CDI, MacArthur Bates Communicative Development Inventories.

Supplementary Figure 12. Effects of rising daily air temperatures on MB-CDI scores with heat thresholds set at the 85th percentile

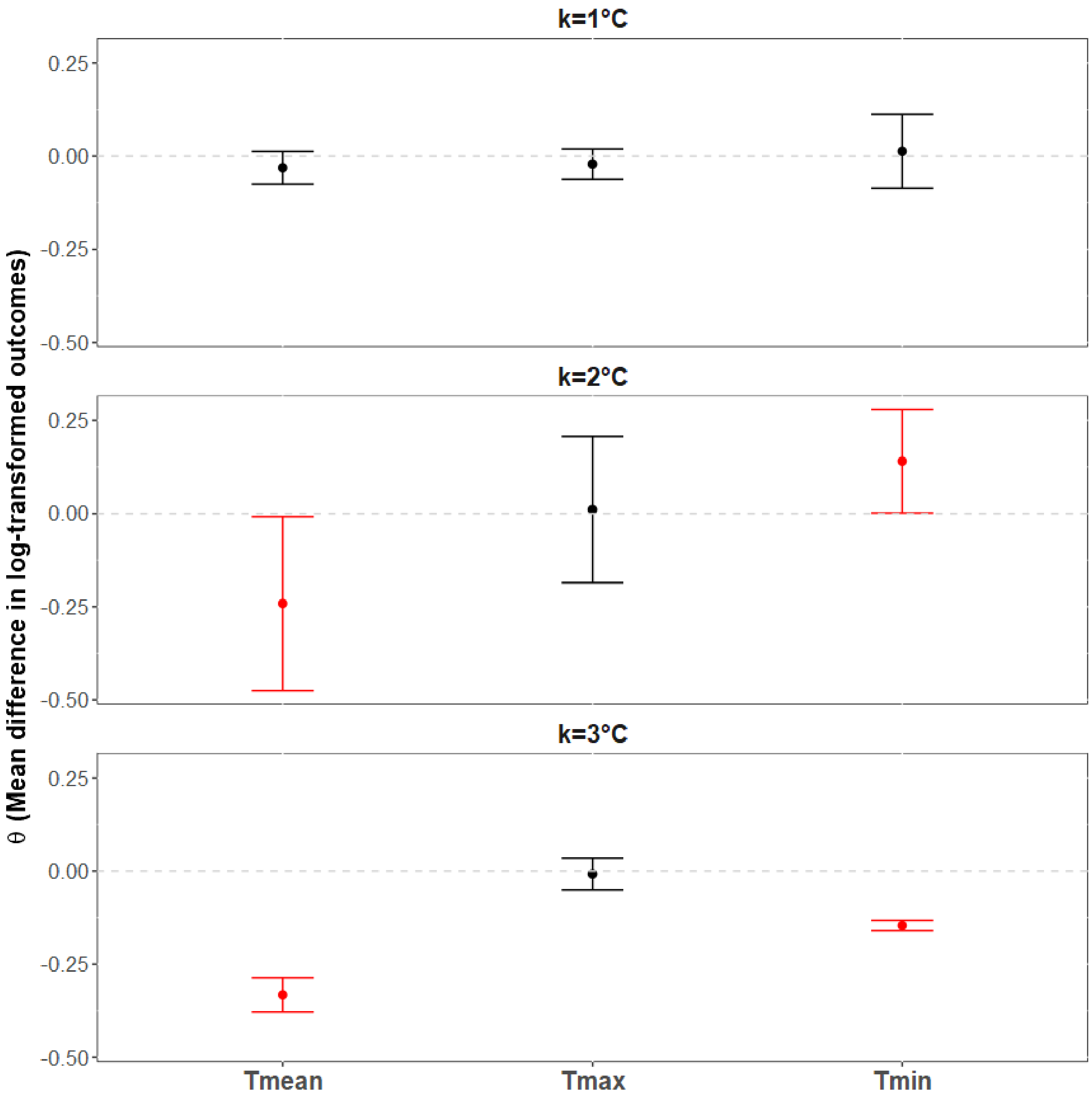

X-axis: Temperature indicators; Y-axis: Mean difference in log-transformed outcomes.
Mean differences in log-transformed MB-CDI scores are plotted along with 95% confidence intervals for counterfactual scenarios involving increases in daily temperature by 1, 2, and 3°C, with heat thresholds set at the 85th percentile for overall (Tmean=19.3°C), daytime (Tmax=25.9°C), and night-time (Tmin=14.1°C) temperatures. Estimates shown in red indicate statistical significance.
Legend. MB-CDI, MacArthur Bates Communicative Development Inventories.

References

1. Attig M, Weinert S. What Impacts Early Language Skills? Effects of Social Disparities and Different Process Characteristics of the Home Learning Environment in the First 2 Years. *Frontiers in Psychology*. 2020;11. Accessed January 10, 2024. https://www.frontiersin.org/articles/10.3389/fpsyg.2020.557751

2. Carolan M, Frankowska D. Advanced maternal age and adverse perinatal outcome: A review of the evidence. *Midwifery*. 2011;27(6):793-801. doi:10.1016/j.midw.2010.07.006

3. Zweifel JE, Woodward JT. The risky business of advanced paternal age: neurodevelopmental and psychosocial implications for children of older fathers. *Fertility and Sterility*. 2022;118(6):1013-1021. doi:10.1016/j.fertnstert.2022.10.029

4. D'Onofrio BM, Rickert ME, Frans E, et al. Paternal age at childbearing and offspring psychiatric and academic morbidity. *JAMA Psychiatry*. 2014;71(4):432-438. doi:10.1001/jamapsychiatry.2013.4525

5. Goisis A, Schneider DC, Myrskylä M. The reversing association between advanced maternal age and child cognitive ability: evidence from three UK birth cohorts. *Int J Epidemiol*. 2017;46(3):850-859. doi:10.1093/ije/dyw354

6. Kacenelenbogen N, Dramaix-Wilmet M, Schetgen M, Roland M, Godin I. Parental separation: a risk for the psychomotor development of children aged 28 to 32 months? A cross-sectional study. *BMC Pediatr*. 2016;16(1):89. doi:10.1186/s12887-016-0621-y

7. Clarke-Stewart KA, Vandell DL, McCartney K, Owen MT, Booth C. Effects of parental separation and divorce on very young children. *Journal of Family Psychology*. 2000;14(2):304-326. doi:10.1037/0893-3200.14.2.304

8. Smith NR, Kelly YJ, Nazroo JY. Ethnic differences in cognitive development in the first 7 years: does maternal generational status matter? *J Epidemiol Community Health*. 2016;70(5):506-512. doi:10.1136/jech-2015-205864

9. Insee. Ethnic-based statistics. September 16, 2016. Accessed November 1, 2023. https://www.insee.fr/en/information/2388586

10. Adélaïde L, Hough I, Seyve E, et al. Environmental and social inequities in continental France: an analysis of exposure to heat, air pollution, and lack of vegetation. *J Expo Sci Environ Epidemiol*. Published online January 26, 2024. doi:10.1038/s41370-024-00641-6

11. Benz SA, Burney JA. Widespread Race and Class Disparities in Surface Urban Heat Extremes Across the United States. *Earth's Future*. 2021;9(7):e2021EF002016. doi:10.1029/2021EF002016

12. Klebanov PK, Brooks-Gunn J, Duncan GJ. Does Neighborhood and Family Poverty Affect Mothers' Parenting, Mental Health, and Social Support? *Journal of Marriage and Family*. 1994;56(2):441-455. doi:10.2307/353111

13. Pornet C, Delpierre C, Dejardin O, et al. Construction of an adaptable European transnational ecological deprivation index: the French version. *J Epidemiol Community Health*. 2012;66(11):982-989. doi:10.1136/jech-2011-200311

14. Boukhabl M, Alkam D. Impact of Vegetation on Thermal Conditions Outside, Thermal Modeling of Urban Microclimate, Case Study: The Street of the Republic, Biskra. *Energy Procedia*. 2012;18:73-84. doi:10.1016/j.egypro.2012.05.019

15. Dadvand P, Nieuwenhuijsen MJ, Esnaola M, et al. Green spaces and cognitive development in primary schoolchildren. *Proc Natl Acad Sci U S A*. 2015;112(26):7937-7942. doi:10.1073/pnas.1503402112

16. Dockx Y, Bijnens EM, Luyten L, et al. Early life exposure to residential green space impacts cognitive functioning in children aged 4 to 6 years. *Environ Int*. 2022;161:107094. doi:10.1016/j.envint.2022.107094

17. Grosjean F. *Bilingual: Life and Reality*. Harvard university press; 2010. Accessed August 21, 2024. https://www.degruyter.com/document/doi/10.4159/9780674056459-intro/html

18. Bjerkedal T, Kristensen P, Skjeret GA, Brevik JI. Intelligence test scores and birth order among young Norwegian men (conscripts) analyzed within and between families. *Intelligence*. 2007;35(5):503-514. doi:10.1016/j.intell.2007.01.004

19. Guo T, Wang Y, Zhang H, et al. The association between ambient temperature and the risk of preterm birth in China. *Sci Total Environ*. 2018;613-614:439-446. doi:10.1016/j.scitotenv.2017.09.104

20. Shin EK, LeWinn K, Bush N, Tylavsky FA, Davis RL, Shaban-Nejad A. Association of Maternal Social Relationships With Cognitive Development in Early Childhood. *JAMA Netw Open*. 2019;2(1):e186963. doi:10.1001/jamanetworkopen.2018.6963

21. Tong L, Kalish BT. The impact of maternal obesity on childhood neurodevelopment. *J Perinatol*. 2021;41(5):928-939. doi:10.1038/s41372-020-00871-0

22. Chen H, Qin L, Gao R, et al. Neurodevelopmental effects of maternal folic acid supplementation: a systematic review and meta-analysis. *Critical Reviews in Food Science and Nutrition*. 2023;63(19):3771-3787. doi:10.1080/10408398.2021.1993781

23. Key APF, Ferguson M, Molfese DL, Peach K, Lehman C, Molfese VJ. Smoking during Pregnancy Affects Speech-Processing Ability in Newborn Infants. *Environmental Health Perspectives*. 2007;115(4):623-629. doi:10.1289/ehp.9521

24. Gilman SE, Gardener H, Buka SL. Maternal Smoking during Pregnancy and Children's Cognitive and Physical Development: A Causal Risk Factor? *American Journal of Epidemiology*. 2008;168(5):522-531. doi:10.1093/aje/kwn175

25. Lassen K, Oei TPS. Effects of maternal cigarette smoking during pregnancy on long-term physical and cognitive parameters of child development. *Addictive Behaviors*. 1998;23(5):635-653. doi:10.1016/S0306-4603(98)00022-7

26. Hendricks G, Malcolm-Smith S, Adnams C, Stein DJ, Donald KAM. Effects of prenatal alcohol exposure on language, speech and communication outcomes: a review longitudinal studies. *Acta Neuropsychiatrica*. 2019;31(2):74-83. doi:10.1017/neu.2018.28

27. Galéra C, Bernard JY, van der Waerden J, et al. Prenatal Caffeine Exposure and Child IQ at Age 5.5 Years: The EDEN Mother-Child Cohort. *Biol Psychiatry*. 2016;80(9):720-726. doi:10.1016/j.biopsych.2015.08.034

28. Berglundh S, Vollrath M, Brantsæter AL, et al. Maternal caffeine intake during pregnancy and child neurodevelopment up to eight years of age—Results from the Norwegian Mother, Father and Child Cohort Study. *Eur J Nutr*. 2021;60(2):791-805. doi:10.1007/s00394-020-02280-7

29. Oken E, Radesky JS, Wright RO, et al. Maternal Fish Intake during Pregnancy, Blood Mercury Levels, and Child Cognition at Age 3 Years in a US Cohort. *American Journal of Epidemiology*. 2008;167(10):1171-1181. doi:10.1093/aje/kwn034

30. Daniels JL, Longnecker MP, Rowland AS, Golding J, Health TASTU of BI of C. Fish Intake During Pregnancy and Early Cognitive Development of Offspring. *Epidemiology*. 2004;15(4):394. doi:10.1097/01.ede.0000129514.46451.ce

31. Huang Y, Iosif AM, Hansen RL, Schmidt RJ. Maternal polyunsaturated fatty acids and risk for autism spectrum disorder in the MARBLES high-risk study. *Autism*. 2020;24(5):1191-1200. doi:10.1177/1362361319877792

32. Horta BL, de Sousa BA, de Mola CL. Breastfeeding and neurodevelopmental outcomes. *Current Opinion in Clinical Nutrition and Metabolic Care*. 2018;21(3):174-178. doi:10.1097/MCO.0000000000000453

33. Leaper C, Smith TE. A meta-analytic review of gender variations in children's language use: talkativeness, affiliative speech, and assertive speech. *Dev Psychol*. 2004;40(6):993-1027. doi:10.1037/0012-1649.40.6.993

34. Fiore AM, Naik V, Leibensperger EM. Air Quality and Climate Connections. *Journal of the Air & Waste Management Association*. 2015;65(6):645-685. doi:10.1080/10962247.2015.1040526

35. Buckley JP, Samet JM, Richardson DB. Commentary: Does Air Pollution Confound Studies of Temperature? *Epidemiology*. 2014;25(2):242. doi:10.1097/EDE.0000000000000051

36. Guilbert A, Bernard JY, Peyre H, et al. Prenatal and childhood exposure to ambient air pollution and cognitive function in school-age children: Examining sensitive windows and sex-specific associations. *Environ Res*. 2023;235:116557. doi:10.1016/j.envres.2023.116557

37. Hoff-Ginsberg E, Shatz M. Linguistic input and the child's acquisition of language. *Psychological Bulletin*. 1982;92(1):3-26. doi:10.1037/0033-2909.92.1.3

38. Madigan S, McArthur BA, Anhorn C, Eirich R, Christakis DA. Associations Between Screen Use and Child Language Skills: A Systematic Review and Meta-analysis. *JAMA Pediatrics*. 2020;174(7):665-675. doi:10.1001/jamapediatrics.2020.0327

39. Dionne G, Touchette E, Forget-Dubois N, et al. Associations Between Sleep-Wake Consolidation and Language Development in Early Childhood: A Longitudinal Twin Study. *Sleep*. 2011;34(8):987-995. doi:10.5665/SLEEP.1148

40. Vissers C, Koolen S. Theory of Mind Deficits and Social Emotional Functioning in Preschoolers with Specific Language Impairment. *Frontiers in Psychology*. 2016;7. Accessed January 16, 2024. https://www.frontiersin.org/articles/10.3389/fpsyg.2016.01734

41. Bretherton L, Prior M, Bavin E, Cini E, Eadie P, Reilly S. Developing relationships between language and behaviour in preschool children from the Early Language in Victoria Study: implications for intervention. *Emotional and Behavioural Difficulties*. 2014;19(1):7-27. doi:10.1080/13632752.2013.854956

42. Longobardi E, Spataro P, Frigerio A, Rescorla L. Language and social competence in typically developing children and late talkers between 18 and 35 months of age. *Early Child Development and Care*. 2016;186(3):436-452. doi:10.1080/03004430.2015.1039529

43. McCabe PC. Social and behavioral correlates of preschoolers with specific language impairment. *Psychology in the Schools*. 2005;42(4):373-387. doi:10.1002/pits.20064

44. Williams SM, Farmer VL, Taylor BJ, Taylor RW. Do More Active Children Sleep More? A Repeated Cross-Sectional Analysis Using Accelerometry. *PLOS ONE*. 2014;9(4):e93117. doi:10.1371/journal.pone.0093117

45. Chang Z, Lei W. A Study on the Relationship Between Physical Activity, Sedentary Behavior, and Sleep Duration in Preschool Children. *Frontiers in Public Health*. 2021;9. Accessed January 16, 2024. https://www.frontiersin.org/articles/10.3389/fpubh.2021.618962

46. Barnett MA, Gustafsson H, Deng M, Mills-Koonce WR, Cox M. Bidirectional Associations Among Sensitive Parenting, Language Development, and Social Competence. *Infant and child development*. 2012;21(4):374. doi:10.1002/icd.1750

47. Dale PS, Tosto MG, Hayiou-Thomas ME, Plomin R. Why does parental language input style predict child language development? A twin study of gene–environment correlation. *J Commun Disord*. 2015;57:106-117. doi:10.1016/j.jcomdis.2015.07.004

48. Wang L, Di J, Wang Q, et al. Heat exposure induced risks of preterm birth mediated by maternal hypertension. *Nat Med*. 2024;30(7):1974-1981. doi:10.1038/s41591-024-03002-w

49. Willi Y, Van Buskirk J. A review on trade-offs at the warm and cold ends of geographical distributions. *Philosophical Transactions of the Royal Society B: Biological Sciences*. 2022;377(1848):20210022. doi:10.1098/rstb.2021.0022

50. Barbalat G, Guilbert A, Adelaïde L, et al. Impact of early life exposure to heat and cold on linguistic development in two-year-old children: findings from the ELFE cohort study. *Environ Health*. 2025;24(1):19. doi:10.1186/s12940-025-01173-8
